# Whole-Grain Staple Replacement and Cardiometabolic Phenotypes in Adults at High Risk of Type 2 Diabetes: An Extended Randomized-Trial Analysis with NHANES and Country-Level Context

**DOI:** 10.3390/nu18172758

**Published:** 2026-08-23

**Authors:** Weihua Dong, Yongjun Wang, Ziyuan Liu, Lingling Ou, Qin Zhuo, Zhaolong Gong, Tingting Liu

**Affiliations:** Key Laboratory of Public Nutrition and Health of National Health Commission, National Institute for Nutrition and Health, Chinese Center for Disease Control and Prevention & Chinese Academy of Preventive Medicine, Beijing 100050, China; dwh19861535785@163.com (W.D.); gongzl@ninh.chinacdc.cn (Z.G.)

**Keywords:** whole grains, staple food replacement, prediabetes, glucose–lipid metabolism, adiposity, triglyceride–glucose index

## Abstract

Background/Objectives: Adults at high risk of type 2 diabetes frequently show concurrent dyslipidemia, insulin resistance, and central adiposity before overt diabetes develops. We evaluated cardiometabolic responses to standardized whole-grain staple replacement and examined grain-quality patterns in free-living and country-level settings. Methods: This secondary exploratory analysis included 144 participants (48 per group) from a 12-week, three-arm randomized dietary trial. Participants were analyzed according to their randomized assignments to 100 g/day, 50 g/day, or control. Lipid, anthropometric, body composition, and composite cardiometabolic phenotypes were evaluated. Longitudinal and time-weighted cumulative effects were estimated using linear mixed-effects and regression models, with Benjamini–Hochberg false discovery rate (FDR) correction applied within the corresponding multiplicity domains. Complementary analyses included 30,769 NHANES adults without diabetes, including 12,169 with prediabetes, and descriptive country-level data on whole-grain intake, high-FPG-attributable diabetes burden, and cereal supply. Results: Within the extended phenotype panel, HWG versus control time-weighted cumulative effects included triglycerides (β = −0.220, 95% CI −0.397 to −0.043; FDR-adjusted *p* = 0.047), the atherogenic index of plasma (β = −0.125, −0.191 to −0.060; FDR-adjusted *p* = 0.002), and the triglyceride–glucose index (β = −0.301, −0.429 to −0.173; FDR-adjusted *p* < 0.001). Conventional lipid components showed distinct temporal responses. In NHANES, higher whole-grain exposure and more favorable grain-quality ratios were associated with lower adiposity- and insulin resistance-related phenotypes, whereas refined-grain exposure generally showed the opposite pattern. Country-level analyses showed marked heterogeneity in whole-grain intake, diabetes burden, and cereal supply context. Conclusions: Standardized whole-grain staple replacement was associated with short-term changes across several cardiometabolic dimensions in adults at high risk of type 2 diabetes, with the clearest extended-phenotype signals involving triglyceride-related and glucose–triglyceride coupling measures. NHANES identified cross-sectional grain-quality associations in free-living adults; country-level analyses described heterogeneity in whole-grain intake, diabetes burden, and cereal supply context.

## 1. Introduction

Diabetes high-risk states encompass a broader metabolic risk profile characterized by central adiposity, insulin resistance, atherogenic dyslipidemia, and clustering of other cardiometabolic risk factors [1]. In 2021, approximately 529 million people worldwide had diabetes, and this number is projected to exceed 1.31 billion by 2050. Higher fasting plasma glucose has become an important metabolic risk factor for the global burden of non-communicable diseases [2,3]. Its harms are also associated with increased risks of all-cause mortality and cardiovascular disease, with diabetes and elevated fasting plasma glucose linked to multiple vascular outcomes [4,5]. At this stage, elevated triglycerides, reduced high-density lipoprotein cholesterol (HDL-C), abdominal adiposity, and abnormal body fat distribution often coexist with dysglycemia, reflecting the intersection of increased hepatic lipid output, lipoprotein remodeling, visceral adipose dysfunction, and insulin resistance [6,7]. The INTERHEART study also showed that dyslipidemia, abdominal adiposity, and diabetes are important modifiable risk factors for myocardial infarction [8]. Assessment of populations with glycemic risk should therefore encompass both glycemic abnormalities and the broader constellation of cardiometabolic risk.

Abnormal glucose regulation commonly develops alongside remodeling of lipid composition, insulin sensitivity, abdominal fat accumulation, and body fat distribution, creating interrelated cardiometabolic phenotypes before overt clinical disease is established. AIP, TyG, METS-IR, VAI, CVAI, LAP, and related indices integrate triglyceride–HDL-C composition, glucose–triglyceride coupling, obesity, and visceral adiposity information and therefore describe distinct dimensions within this metabolic risk structure [9,10,11,12,13,14,15,16,17,18,19,20]. Extending intervention assessment beyond isolated laboratory measures can clarify whether changes in staple-food quality are accompanied by coordinated responses across these early cardiometabolic dimensions.

Whole grains are central to dietary strategies aimed at improving carbohydrate quality. Global Burden of Disease analyses identify low whole-grain intake as an important dietary risk factor, while higher whole-grain and dietary fiber intake are associated with lower risks of type 2 diabetes, cardiovascular disease, and all-cause mortality [21,22,23]. Whole grains retain bran, germ, dietary fiber, and a less disrupted food matrix than refined grains, with potential effects on glycemic handling, satiety, and fermentation-related metabolic pathways [24,25]. The translational relevance of these effects depends partly on staple-food form. In many Asian dietary settings, habitual grain intake is centered on familiar rice- and wheat-based staples, so the feasibility of whole-grain replacement may depend on whether these foods can be incorporated into existing meal structures [26,27,28].

Randomized evidence in populations with elevated glycemic risk has often centered on individual glucose or lipid endpoints, leaving the broader cardiometabolic response to standardized staple replacement less well characterized. This gap is particularly relevant when metabolic risk is already distributed across triglyceride-rich lipoprotein metabolism, insulin resistance, abdominal adiposity, and body fat distribution. Population dietary data can further show whether comparable grain-quality patterns occur under free-living conditions, while country-level information can locate these patterns within substantially different cereal consumption and supply backgrounds.

This secondary exploratory analysis used data from a 12-week, three-arm randomized dietary intervention to examine longitudinal changes in glucose–lipid metabolism, anthropometry, body fat distribution, and composite cardiometabolic phenotypes after standardized whole-grain staple replacement in adults at high risk of type 2 diabetes. NHANES was subsequently used to evaluate associations between habitual grain quality and cardiometabolic phenotypes in free-living adults, and harmonized country-level data were used to describe the joint distribution of whole-grain intake, high-FPG-attributable diabetes burden, and cereal supply context.

## 2. Materials and Methods

### 2.1. Study Population and Data Sources

An overview of participant flow in the randomized trial and analytic sample selection for the NHANES and country-level analyses is provided in Figure S1.

#### 2.1.1. Randomized Controlled Dietary Intervention

##### Study Design and Participants

The RCT component of the present study was a secondary exploratory analysis of data from a 12-week, three-arm, parallel randomized controlled dietary intervention in adults at high risk of type 2 diabetes. The trial included repeated metabolic assessments at baseline (week 0), week 6, and week 12, which were used in the present analysis to evaluate fasting glucose, together with lipid, anthropometric, body composition, and composite cardiometabolic phenotypes. A previous report from this randomized trial examined serum 25-hydroxyvitamin D responses to the intervention [29]; the present analysis addresses a distinct secondary question focused on cardiometabolic phenotypes. The study protocol was approved by the Ethics Committee of the National Institute for Nutrition and Health, Chinese Center for Disease Control and Prevention (approval number 2023-002; 21 June 2023) and prospectively registered with the Chinese Clinical Trial Registry (ChiCTR2300072952; 28 June 2023). All participants provided written informed consent before enrollment.

Participants were local adults aged 18–65 years with features of high risk for type 2 diabetes or prediabetes. Eligibility required either fasting plasma glucose (FPG) of 5.6–6.9 mmol/L or hemoglobin A1c (HbA1c) of 5.7–6.4%, according to the ADA criteria applied during study screening [30]. Exclusion criteria included diagnosed diabetes, gastrointestinal dysfunction, malignant tumors, cardiovascular or cerebrovascular disease, severe hepatic or renal dysfunction, severe infectious or autoimmune disease, current use of glucose-lowering, lipid-lowering, or antihypertensive medications, pregnancy, planned pregnancy or lactation, continuous use of nutritional supplements within the previous month, allergy to whole grains, baseline whole-grain intake exceeding 50 g/day, weight change greater than 5% in the previous 3 months, or current use of medications that could affect body weight.

##### Randomization and Dietary Intervention

Eligible participants were randomly assigned in a 1:1:1 ratio to the high-dose whole-grain group (HWG, 100 g/day), the medium-dose whole-grain group (MWG, 50 g/day), or the control group (CG). The randomization sequence was generated by an independent statistician using a computer-generated block randomization procedure, and allocation concealment was implemented using sequentially numbered, opaque, sealed envelopes. Because of the nature of the dietary intervention, participants and field staff could not be blinded; however, laboratory personnel and statistical analysts remained blinded to group assignment until the completion of the main analyses.

The present analysis included 144 participants from the glycemic risk cohort of the registered 12-week randomized dietary trial, with 48 participants in each of the HWG, MWG, and CG groups. This secondary analysis focused on an extended panel of cardiometabolic phenotypes, with FPG included as a directly measured glycemic indicator. Participants were analyzed according to their original randomized assignments, and all available repeated measurements contributed to the longitudinal analyses.

The HWG and MWG groups received standardized whole-grain staple foods providing daily whole-grain targets of 100 and 50 g, respectively. These targets represented the whole-grain equivalents supplied through the study foods, while the corresponding finished-product amounts varied according to formulation. The intervention foods were provided as commonly consumed wheat- and rice-based staple preparations, with formulation-specific differences in whole-grain composition and proportions used to achieve the assigned doses. The rice-based formulations incorporated combinations of brown or red rice with other whole grains, whereas the wheat-based formulations used whole-grain wheat ingredients. Study foods were manufactured by Tongfu Group Co., Ltd. (Shijiazhuang, China), transported under cold-chain conditions, distributed every 2 weeks, and reheated before consumption. Product formulations and nutritional characteristics are summarized in Table S1. Participants in the CG maintained their habitual dietary pattern during the 12-week intervention and received the corresponding whole-grain foods after study completion.

##### Dietary and Lifestyle Assessment

Study-food use was documented using daily intake records, participant-held electronic scales, biweekly collection of records, and weighing of remaining foods before subsequent distribution. Investigators maintained regular telephone and WeChat contact with participants throughout the intervention. All three groups received the same healthy diet and lifestyle education materials. Dietary assessments at baseline and during the intervention were used to characterize background whole-grain and refined-grain intake. To derive a target-based estimate of total whole-grain exposure during the intervention, background whole-grain intake from the dietary assessment was combined with the randomized study-food whole-grain-equivalent target.

Questionnaires, physical measurements, and biological samples were collected at baseline, week 6, and week 12. Questionnaire assessments obtained information on sociodemographic characteristics, lifestyle, previous health status, and dietary intake. Marital status was categorized as married or other (unmarried, divorced, widowed, etc.); occupation was categorized as white-collar, blue-collar, or unemployed, with white-collar work mainly referring to managerial, professional, technical, or office-based work, and blue-collar work mainly referring to manual labor, production processing, or service-related physical work. Alcohol drinking status was classified as non-drinking or current drinking based on baseline self-report; current drinking was defined as alcohol consumption during the previous 3 months. Sufficient physical activity was defined as at least 150 min/week of moderate-intensity physical activity or 75 min/week of vigorous-intensity physical activity [31]. Medication use and major health events were recorded during follow-up.

##### Anthropometric and Body Composition Assessment

Physical measurements included height, weight, waist circumference, hip circumference, and body composition. Waist circumference was measured at the midpoint between the lower costal margin and the iliac crest at the end of a normal expiration. Body composition was assessed using an InBody770 multifrequency bioelectrical-impedance analyzer (InBody Co., Ltd., Seoul, Republic of Korea). Standardized premeasurement conditions addressed recent food and beverage intake, alcohol, exercise, bathing, bladder emptying, footwear, and metal accessories to reduce short-term variation related to hydration and body-fluid distribution. Body mass index (BMI) was calculated as weight (kg) divided by height squared (m^2^). All investigators received standardized training and completed field assessments according to standardized procedures.

##### Biochemical Measurements

All laboratory indicators were measured using fasting venous blood collected in the morning after an 8–12 h overnight fast. Blood samples were centrifuged, aliquoted, and stored according to standardized procedures and then transported to the central laboratory of the National Institute for Nutrition and Health, Chinese Center for Disease Control and Prevention & Chinese Academy of Preventive Medicine, for centralized analysis. FPG, TC, TG, HDL-C, and LDL-C were measured using enzymatic methods on a Hitachi 7600 automated biochemical analyzer (Hitachi High-Tech, Tokyo, Japan), with LDL-C determined by a direct assay. Laboratory measurements were performed using standardized commercial reagents under the central laboratory quality-control procedures.

#### 2.1.2. NHANES Nationally Representative Population

The NHANES analysis was used to evaluate cross-sectional associations between dietary grain quality and cardiometabolic indicators among free-living adults in the United States. Data from seven cycles from 2005–2006 to the 2017–March 2020 prepandemic cycle were combined; the 2017–March 2020 prepandemic file was handled according to NCHS analytic guidance, and the 2017–2018 single cycle was not included again in the main analysis. NHANES uses a complex, multistage probability sampling design; therefore, all analyses incorporated the masked variance pseudo-strata, masked variance pseudo-primary sampling units, and the sample weight corresponding to the analytic domain required for each model. Multi-cycle weights were rescaled according to the number of years represented by each cycle: 2.0 years for each regular 2-year cycle from 2005–2006 to 2015–2016 and 3.2 years for the 2017–March 2020 prepandemic file, divided by 15.2 total represented years. In the current primary analysis, survey weights were selected according to the analytic domain required for each exposure–outcome model. Models for dietary grain-quality exposures and non-fasting outcomes were analyzed within the dietary-recall weight domain, whereas models involving fasting biochemical outcomes or fasting-derived composite indicators were restricted to the fasting subsample and used the corresponding pooled fasting subsample weights. All pooled weights were constructed using the same multi-cycle rescaling approach to maintain consistency between the survey cycle structure, analytic sample, outcome ascertainment, and NHANES complex survey design [32].

The analysis was restricted to adults aged 20 years or older, excluding pregnant participants, participants with invalid dietary data, and participants with diabetes. Diabetes was defined by any of the following criteria: self-reported physician diagnosis of diabetes, use of glucose-lowering medication, HbA1c ≥ 6.5%, or FPG ≥ 126 mg/dL; the FPG criterion was applied only to participants with fasting glucose measurements. The prediabetes subgroup was further defined within the main analytic sample by HbA1c 5.7–6.4% or FPG 100–125 mg/dL [30]. Outcomes related to fasting biochemical indicators were analyzed within the fasting subsample weight domain, whereas dietary exposures, model covariates, and non-fasting outcomes were handled within the corresponding dietary-recall weight domain.

Dietary data validity was defined based on dietary recall quality control and completeness of the core FPED-derived grain exposure variables. Included participants were required to have reliable dietary recall information and complete FPED-derived whole-grain, refined-grain, total-grain, and total energy variables in the analytic database. The main analytic database did not exclude additional participants based on extreme energy-intake thresholds. To evaluate the influence of extreme exposure values on model estimates, winsorized exposure variables were prespecified for sensitivity analyses.

#### 2.1.3. Country-Level Contextual Analysis

The country-level analysis used the country-year as the unit of analysis to describe the joint distribution of whole-grain intake, high-FPG-attributable diabetes burden, and cereal supply context. Country-level whole-grain intake was obtained from the Global Dietary Database (GDD). Age-standardized DALY rates for diabetes attributable to high fasting plasma glucose were obtained from the Global Burden of Disease (GBD) study, and cereal supply was obtained from FAOSTAT Food Balance Sheets using food supply quantity for cereals excluding beer. SDI, SDI categories, GBD regions, and super-regions were linked through GBD location identifiers [33,34,35].

Analysis years were restricted to common years for which the three core variables from GDD, GBD, and FAOSTAT could be matched. During data integration, country names, ISO3/M49 codes, GBD location identifiers, and common aliases were harmonized first, followed by checks for duplicate records, missing core variables, and consistency of statistical scopes.

### 2.2. Metabolic Phenotype Indicator System and Formula Construction

Glucose–lipid metabolic indicators were defined using consistent indicator definitions, unit conversions, and composite formulas. The RCT analysis covered directly measured glucose and lipid indicators, anthropometric measures, body composition outputs, and composite indicators derived from glucose, lipid, and body shape variables. NHANES analyses evaluated cross-sectional associations between habitual grain quality and available cardiometabolic phenotypes.

#### 2.2.1. Unit Conversion

To ensure consistent indicator calculation between the RCT and NHANES, lipid and glucose variables required in mg/dL by the formulas were converted before calculation using the following coefficients:

$${\mathrm{TG}}_{\mathrm{mg}/\mathrm{dL}}={\mathrm{TG}}_{\mathrm{mmol}/L}\times 88.57$$

$${\mathrm{TC}}_{\mathrm{mg}/\mathrm{dL}}={\mathrm{TC}}_{\mathrm{mmol}/L}\times 38.67$$

$${\mathrm{HDL}\text{-}C}_{\mathrm{mg}/\mathrm{dL}}={\mathrm{HDL}\text{-}C}_{\mathrm{mmol}/L}\times 38.67$$

$${\mathrm{FPG}}_{\mathrm{mg}/\mathrm{dL}}={\mathrm{FPG}}_{\mathrm{mmol}/L}\times 18.018$$

#### 2.2.2. Basic Body Shape Indicators

Basic body shape indicators included BMI [36], waist-to-height ratio (WHtR) [37], and waist circumference (WC).

$$\mathrm{BMI}=\frac{{\mathrm{weight}}_{\mathrm{kg}}}{{\mathrm{height}}_{m}^{2}}$$

$$\mathrm{WHtR}=\frac{\mathrm{WC}}{\mathrm{height}}$$

#### 2.2.3. Glucose–Lipid Metabolic Composite Indicators

AIP [38], the TG/HDL-C ratio, and TyG [11] were calculated using the following formulas.

$$\mathrm{AIP}=\log_{10}\left(\frac{{\mathrm{TG}}_{\mathrm{mmol}/L}}{{\mathrm{HDL}\text{-}C}_{\mathrm{mmol}/L}}\right)$$

$$\mathrm{TG}/\mathrm{HDL}\text{-}C=\frac{{\mathrm{TG}}_{\mathrm{mmol}/L}}{{\mathrm{HDL}\text{-}C}_{\mathrm{mmol}/L}}$$

$$\mathrm{TyG}=\ln\left(\frac{{\mathrm{TG}}_{\mathrm{mg}/\mathrm{dL}}\times {\mathrm{FPG}}_{\mathrm{mg}/\mathrm{dL}}}{2}\right)$$

#### 2.2.4. Body Shape, Lipid, and Visceral Adiposity-Related Composite Indicators

The cardiometabolic index (CMI) [39], lipid accumulation product (LAP) [18], VAI [16], Chinese visceral adiposity index (CVAI) [40], METS-IR [14], ABSI [20], body roundness index (BRI) [19], and weight-adjusted waist index (WWI) [41] were calculated according to published formulas.

$$\mathrm{CMI}=\mathrm{WHtR}\times \frac{{\mathrm{TG}}_{\mathrm{mmol}/L}}{{\mathrm{HDL}\text{-}C}_{\mathrm{mmol}/L}}$$

$${\mathrm{LAP}}_{\mathrm{men}}=\left({\mathrm{WC}}_{\mathrm{cm}}-65\right)\times {\mathrm{TG}}_{\mathrm{mmol}/L}$$

$${\mathrm{LAP}}_{\mathrm{women}}=\left({\mathrm{WC}}_{\mathrm{cm}}-58\right)\times {\mathrm{TG}}_{\mathrm{mmol}/L}$$

$${\mathrm{VAI}}_{\mathrm{men}}=\frac{\mathrm{WC}}{39.68+1.88\times \mathrm{BMI}}\times \frac{\mathrm{TG}}{1.03}\times \frac{1.31}{\mathrm{HDL}\text{-}C}$$

$${\mathrm{VAI}}_{\mathrm{women}}=\frac{\mathrm{WC}}{36.58+1.89\times \mathrm{BMI}}\times \frac{\mathrm{TG}}{0.81}\times \frac{1.52}{\mathrm{HDL}\text{-}C}$$

$${CVAI}_{male}=-267.93+0.68\times age+0.03\times BMI+4.00\times WC\;\;\;\;+\;22.00\times {log}_{10}(TG)-16.32\times \mathrm{HDL}\text{-}C$$

$${CVAI}_{female}=-187.32+1.71\times age+4.23\times BMI+1.12\times WC\;\;\;\;+\;39.76\times {log}_{10}(TG)-11.66\times \mathrm{HDL}\text{-}C$$

$$\mathrm{METS}\text{-}\mathrm{IR}=\frac{\ln\left(2\times {\mathrm{FPG}}_{\mathrm{mg}/\mathrm{dL}}+{\mathrm{TG}}_{\mathrm{mg}/\mathrm{dL}}\right)\times \mathrm{BMI}}{\ln\left({\mathrm{HDL}\text{-}C}_{\mathrm{mg}/\mathrm{dL}}\right)}$$

$$\mathrm{ABSI}=\frac{{\mathrm{WC}}_{m}}{{\mathrm{BMI}}^{2/3}\times {\mathrm{height}}_{m}^{1/2}}$$

$$\mathrm{BRI}=364.2-365.5\times \sqrt{1-\frac{{\left(\mathrm{WC}/\left(2\pi \right)\right)}^{2}}{{\left(0.5\times \mathrm{height}\right)}^{2}}}$$

$$\mathrm{WWI}=\frac{{\mathrm{WC}}_{\mathrm{cm}}}{\sqrt{{\mathrm{weight}}_{\mathrm{kg}}}}$$

#### 2.2.5. TyG- and AIP-Derived Indicators

TyG-derived indicators were obtained by multiplying TyG by the corresponding anthropometric or composite adiposity indicator. AIP-derived indicators were obtained by multiplying AIP by the corresponding body shape indicator.

TyG-X=TyG×X

where X ∈ {WC, BMI, WHtR, LAP, CVAI, ABSI, BRI, WWI}.

AIP-X=AIP×X

The triglyceride–cholesterol–body weight index (TCBI) [42] was constructed using the following formula, and the natural logarithm of TCBI was further calculated as log(TCBI) to reduce right skewness. Specifically, TCBI = TG (mg/dL) × TC (mg/dL) × body weight (kg)/1000, and log(TCBI) = ln(TCBI).

$$\log(\mathrm{TCBI})=\ln\left(\frac{{\mathrm{TG}}_{\mathrm{mg}/\mathrm{dL}}\times {\mathrm{TC}}_{\mathrm{mg}/\mathrm{dL}}\times {\mathrm{weight}}_{\mathrm{kg}}}{1000}\right)$$

### 2.3. NHANES Dietary Grain Exposure Definitions

NHANES grain exposures were constructed from dietary interviews and the US Department of Agriculture Food Patterns Equivalents Database (FPED). FPED converts reported foods and beverages into standardized dietary pattern equivalents, with grains expressed as ounce equivalents (oz-eq) and classified as whole grains, refined grains, and total grains [43]. In the following formulas, G_WHOLE, G_REFINED, and G_TOTAL denote individual intakes of whole grains, refined grains, and total grains (oz-eq/day), respectively; energy denotes total energy intake (kcal/day), and fiber denotes dietary fiber intake (g/day).

#### 2.3.1. Whole-Grain Exposure

$$\mathrm{Whole}\text{-}\mathrm{grain}\;\mathrm{density}=\frac{G_{\mathrm{WHOLE}}}{\mathrm{energy}}\times 1000\left(\mathrm{oz}\text{-}\mathrm{eq}/1000\;\mathrm{kcal}\right)$$

$$\mathrm{Whole}\;\mathrm{grain}\;\mathrm{as}\;\mathrm{proportion}\;\mathrm{of}\;\mathrm{total}\;\mathrm{grain}=\frac{G_{\mathrm{WHOLE}}}{G_{\mathrm{TOTAL}}}\times 100\left(\%\right)$$

#### 2.3.2. Grain-Quality Ratio Exposure

$$\mathrm{Whole}\text{-}\mathrm{to}\text{-}\mathrm{refined}\;\mathrm{grain}\;\mathrm{ratio}=\frac{G_{\mathrm{WHOLE}}}{G_{\mathrm{REFINED}}}$$

$$\log\left(1+\mathrm{whole}\text{-}\mathrm{to}\text{-}\mathrm{refined}\;\mathrm{grain}\;\mathrm{ratio}\right)=\;\log1p\left(\frac{G_{\mathrm{WHOLE}}}{G_{\mathrm{REFINED}}}\right)$$

#### 2.3.3. Refined-Grain, Total-Grain, and Dietary Fiber Exposure

$$\mathrm{Refined}\text{-}\mathrm{grain}\;\mathrm{density}=\frac{G_{\mathrm{REFINED}}}{\mathrm{energy}}\times 1000\left(\mathrm{oz}\text{-}\mathrm{eq}/1000\;\mathrm{kcal}\right)$$

$$\mathrm{Total}\text{-}\mathrm{grain}\;\mathrm{density}=\frac{G_{\mathrm{TOTAL}}}{\mathrm{energy}}\times 1000\left(\mathrm{oz}\text{-}\mathrm{eq}/1000\;\mathrm{kcal}\right)$$

$$\mathrm{Dietary}\;\mathrm{fiber}\;\mathrm{density}=\frac{\mathrm{fiber}}{\mathrm{energy}}\times 1000\left(g/1000\;\mathrm{kcal}\right)$$

The supplementary exposure was absolute whole-grain intake (G_WHOLE, oz-eq/day). To reduce the influence of extreme values on ratio- and density-based exposures, winsorization at the 1st–99th percentiles in the main sample was prespecified for whole-grain intake density, the whole-grain/refined-grain ratio, and dietary fiber intake density; winsorized variables were used for sensitivity analyses, whereas the original exposures remained the primary explanatory variables.

### 2.4. Country-Level Descriptive Typology

The country-level descriptive typology used pooled medians of all country-year observations as empirical cut-points for whole-grain intake, high-FPG-attributable diabetes burden, and cereal supply context. Values below the corresponding median were classified as lower, and values at or above the median were classified as higher, providing a common descriptive scale across years and countries.

Type A combined lower whole-grain intake, higher high-FPG-attributable diabetes burden, and higher cereal supply; Type B combined lower whole-grain intake, higher burden, and lower cereal supply; Type C represented lower whole-grain intake and lower burden; Type D represented higher whole-grain intake and higher burden; and Type E represented higher whole-grain intake and lower burden.

### 2.5. Statistical Analysis

Statistical analyses were performed using R 4.5.1 (R Foundation for Statistical Computing, Vienna, Austria) and SAS 9.4 (SAS Institute, Cary, NC, USA). All tests were two-sided. The Benjamini–Hochberg procedure was used to control the false discovery rate (FDR) for analyses involving multiple cardiometabolic phenotypes, with the correction set defined according to the corresponding analysis framework [44].

Continuous variables were reported as means (standard deviations) or medians (first quartile, third quartile) according to their distributions, and categorical variables were reported as counts and percentages. Baseline between-group differences were assessed using analysis of variance, the Kruskal–Wallis test, the χ^2^ test, or Fisher’s exact test according to variable distribution. NHANES estimates of continuous variables and proportions used corresponding survey weights, strata, and PSU for complex survey-weighted estimation; categorical variables were presented as unweighted counts and survey-weighted percentages, between-group differences in continuous variables were assessed using survey-weighted linear regression or rank-transformed models, and categorical variables were assessed using the Rao–Scott-corrected χ^2^ test.

#### 2.5.1. RCT Longitudinal Intervention Effects, Cumulative Changes, and Correlation Structure

Changes from baseline at weeks 6 and 12 were compared between groups, with FDR correction applied within the corresponding analysis framework. The longitudinal RCT analysis used linear mixed-effects models (LMMs), with the original indicator values at baseline, week 6, and week 12 as the dependent variables. Fixed effects included treatment group, time, treatment group × time interaction, age, and sex, with participant specified as a random intercept; CG and baseline were used as references. The models included both baseline and follow-up observations and did not include the baseline value of the same outcome as an additional independent covariate, because baseline measurements were already included within the repeated-measures outcome structure. Participants from the RCT analysis set who missed week 6 or week 12 were not excluded in their entirety; all available repeated observations contributed to likelihood-based model estimation. The main estimates were the MWG versus CG and HWG versus CG group × time interaction terms at week 6 and week 12, reflecting the difference in change from baseline to the corresponding follow-up time point for each intervention group relative to the control group. For the longitudinal RCT analysis, FDR correction was applied jointly to the intervention-by-time contrasts across both follow-up visits and the phenotype panel.

To summarize the overall deviation from baseline during the 12-week trial, we constructed an individual-level normalized trapezoidal time-weighted cumulative change score for each metabolic phenotype. For each participant, changes from baseline to week 6 and week 12 were first calculated. Because the change from baseline was zero at baseline and the two follow-up intervals were of equal duration, the area under the change-from-baseline curve was estimated using the trapezoidal rule. This area was divided by the total follow-up duration to retain the original measurement unit, yielding the following normalized time-weighted cumulative change score, hereafter referred to as the time-weighted cumulative change score:

$$\Delta_{\mathrm{weighted}}=0.5\times \left({\mathrm{value}}_{\mathrm{week}6}-{\mathrm{value}}_{\mathrm{baseline}}\right)+0.25\times \left({\mathrm{value}}_{\mathrm{week}12}-{\mathrm{value}}_{\mathrm{baseline}}\right)$$

This metric was adapted from the time-weighted cumulative exposure approach and was used as a secondary summary measure of longitudinal change in the RCT setting [45]. Unlike a single end-point change, the time-weighted cumulative change score incorporates both interim and final follow-up measurements, thereby providing an integrated estimate of the extent to which each phenotype deviated from baseline during the intervention period. Between-group differences in the time-weighted cumulative change score were estimated using linear regression models adjusted for the baseline value of the corresponding phenotype, age and sex, with contrasts for MWG versus CG, HWG versus CG, and HWG versus MWG. Because this analysis summarizes observed change trajectories, participants were included only when the phenotype values required for the construction of the corresponding score were available. Spearman correlations among time-weighted cumulative changes were used to characterize the correlation structure of the phenotype panel. For time-weighted cumulative effects, FDR correction was applied separately within each pairwise treatment contrast across the phenotype panel.

Sensitivity analyses were restricted to the 134 participants who completed all three scheduled assessments and repeated the longitudinal and time-weighted cumulative models using the same model definitions and FDR correction structures as the main phenotype analyses.

#### 2.5.2. NHANES Cross-Sectional Association and Nonlinear Analysis

Associations between dietary exposures and outcomes in the NHANES cross-sectional data were estimated using survey-weighted linear regression. Each exposure-outcome pair was modeled separately, with adjustment for age, sex, race or ethnicity, survey cycle, education level, family income-to-poverty ratio, smoking status, alcohol drinking status, physical activity status, and total energy intake. Strata, PSU, and weights from the complex sampling design were used for design estimation and variance calculation and were not entered into the regression models as ordinary covariates. The main results were reported as standardized regression coefficients, representing the difference in standardized outcome level associated with each 1-SD increase in exposure. Survey-weighted restricted cubic spline (RCS) regression was further used to assess nonlinear exposure-outcome associations; the number of knots was selected from three, four, and five candidate values according to the Akaike information criterion (AIC), and curves used the exposure median as the reference and were displayed within the 1st to 99th percentile range of the corresponding exposure. Survey-weighted estimation followed NHANES analytic guidance for pooled cycles and domain-specific weights [32], and grain exposures were derived from the USDA FPED framework described above [43]. For the NHANES analyses, FDR correction was applied separately in the main analytic sample and the prediabetes subgroup for each dietary exposure across the evaluated phenotype panel. In the RCS analyses, overall association and nonlinearity *p* values were corrected separately within each exposure.

## 3. Results

### 3.1. Randomized Trial Results

The present RCT analysis comprised 144 participants, with 48 participants in each randomized group. At baseline, the median age was 52.5 years (IQR 43.8–59.0), 64.6% of participants were women, and the median BMI was 24.30 kg/m^2^ (22.25–27.30). Sociodemographic, lifestyle, and routine clinical characteristics were generally similar across the three groups, and biochemical indicators, body composition measures, and composite metabolic risk indicators were also comparable. HDL-C differed in the unadjusted overall comparison, but the overall difference was no longer significant after FDR adjustment. Overall, no consistent baseline imbalance was observed across groups (Table S2).

No serious adverse events clearly related to the study foods were reported during the 12-week follow-up.

Dietary data available for the grain exposure analysis showed clear separation in target-based estimated whole-grain exposure across the randomized groups during the intervention. Median (IQR) target-based estimated total whole-grain exposure, combining background intake with the assigned study-food whole-grain equivalent, was 16.7 (0.0–62.3), 66.7 (50.0–99.0), and 131.8 (115.4–184.0) g/day in the CG, MWG, and HWG groups, respectively. The corresponding median intervention-period refined-grain intake was 188.3 (122.0–235.5), 76.8 (47.9–126.8), and 130.7 (84.3–207.4) g/day. In baseline-adjusted linear models, target-based estimated total whole-grain exposure was higher in MWG than in CG (adjusted mean difference, 43.2 g/day; 95% CI, 8.7–77.8; *p* = 0.014) and in HWG than in CG (110.0 g/day; 95% CI, 76.8–143.2; *p* < 0.001). Refined-grain intake was lower in MWG than in CG (−96.4 g/day; 95% CI, −141.1 to −51.6; *p* < 0.001), whereas the HWG–CG difference was −31.1 g/day (95% CI, −73.3 to 11.1; *p* = 0.148) (Table S3).

At week 12, the FPG change score contrast was −0.350 mmol/L (95% CI −0.692 to −0.008; *p* = 0.045; FDR-adjusted *p* = 0.177) for HWG versus CG and −0.339 mmol/L (95% CI −0.680 to 0.003; *p* = 0.052; FDR-adjusted *p* = 0.194) for MWG versus CG (Table S4).

Conventional lipid components showed distinct temporal responses. HDL-C increased in HWG and decreased in CG, with significant between-group differences at both follow-up visits (week 6, 0.072 ± 0.028 vs. −0.069 ± 0.035; week 12, 0.056 ± 0.026 vs. −0.075 ± 0.034; both FDR-adjusted *p* = 0.023). TC followed a different trajectory: it increased slightly in HWG and decreased in CG at week 6, while by week 12 both groups were below baseline, but the decline remained larger in CG. LDL-C also showed time-dependent between-group effects.

**Figure 1:**
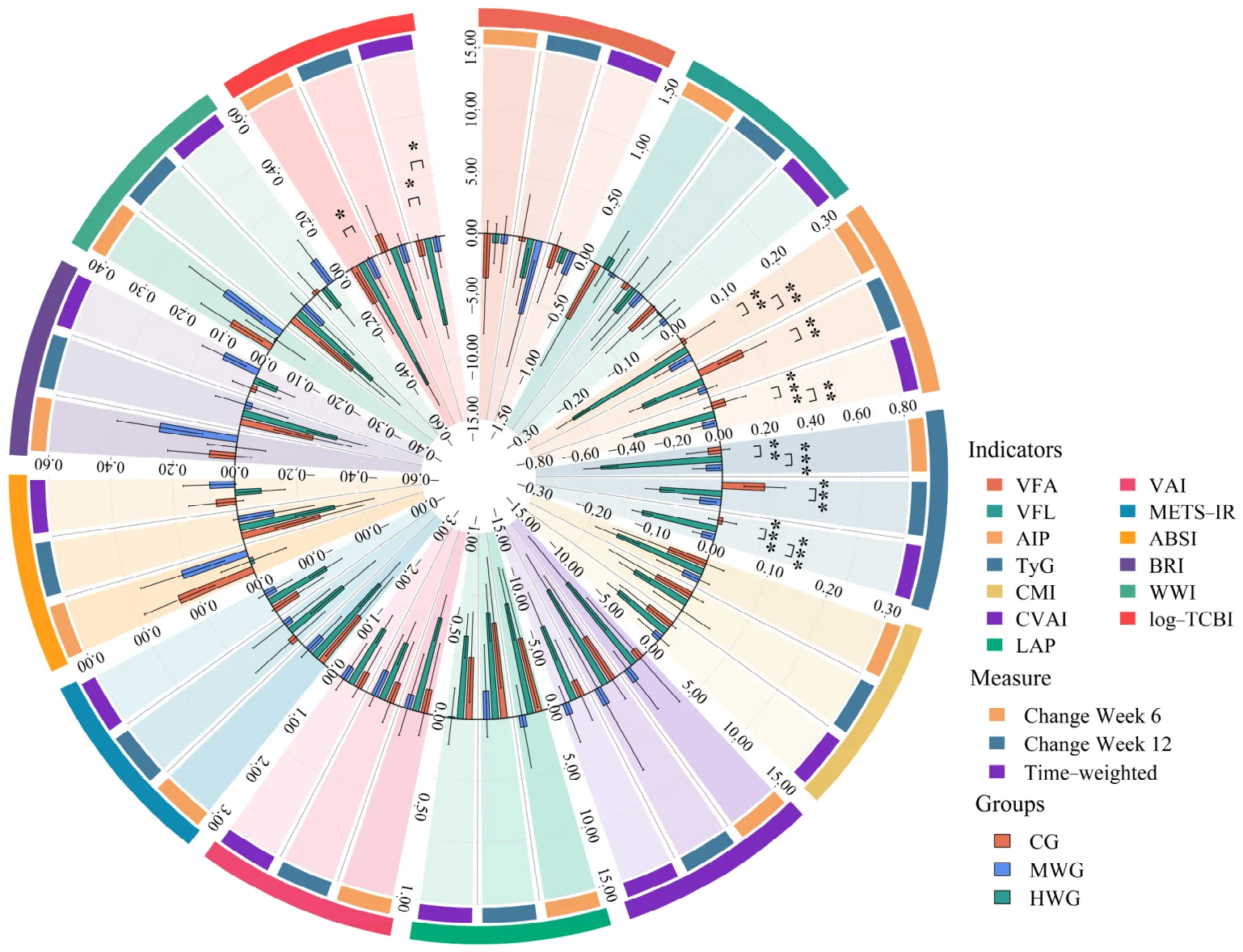
Changes in selected cardiometabolic phenotypes during the randomized intervention. The circular plot shows changes from baseline at week 6 and week 12 and time-weighted cumulative changes for 13 selected cardiometabolic phenotypes across the CG, MWG, and HWG groups. Bars represent pooled group means, with error bars indicating SE. Brackets denote pairwise between-group comparisons, and asterisks indicate significance after FDR correction (* *p* < 0.05, ** *p* < 0.01, *** *p* < 0.001).

Among the derived cardiometabolic phenotypes, intervention-related changes were most evident in AIP and TyG. At week 6, AIP and TyG decreased by 0.214 ± 0.031 and 0.521 ± 0.074 in HWG, respectively, with larger reductions than in CG after FDR adjustment; both phenotypes remained lower than baseline at week 12 (Table S4, Figure 1).

Correlations among cumulative changes showed clustering among phenotypes sharing related metabolic components. AIP correlated closely with TG and TG/HDL-C, TyG-related measures were correlated with several glucose–triglyceride and adiposity-related measures, and visceral adiposity indices showed substantial correlations with waist- and lipid-related measures. The complete correlation matrix is provided in Table S6.

**Figure 2:**
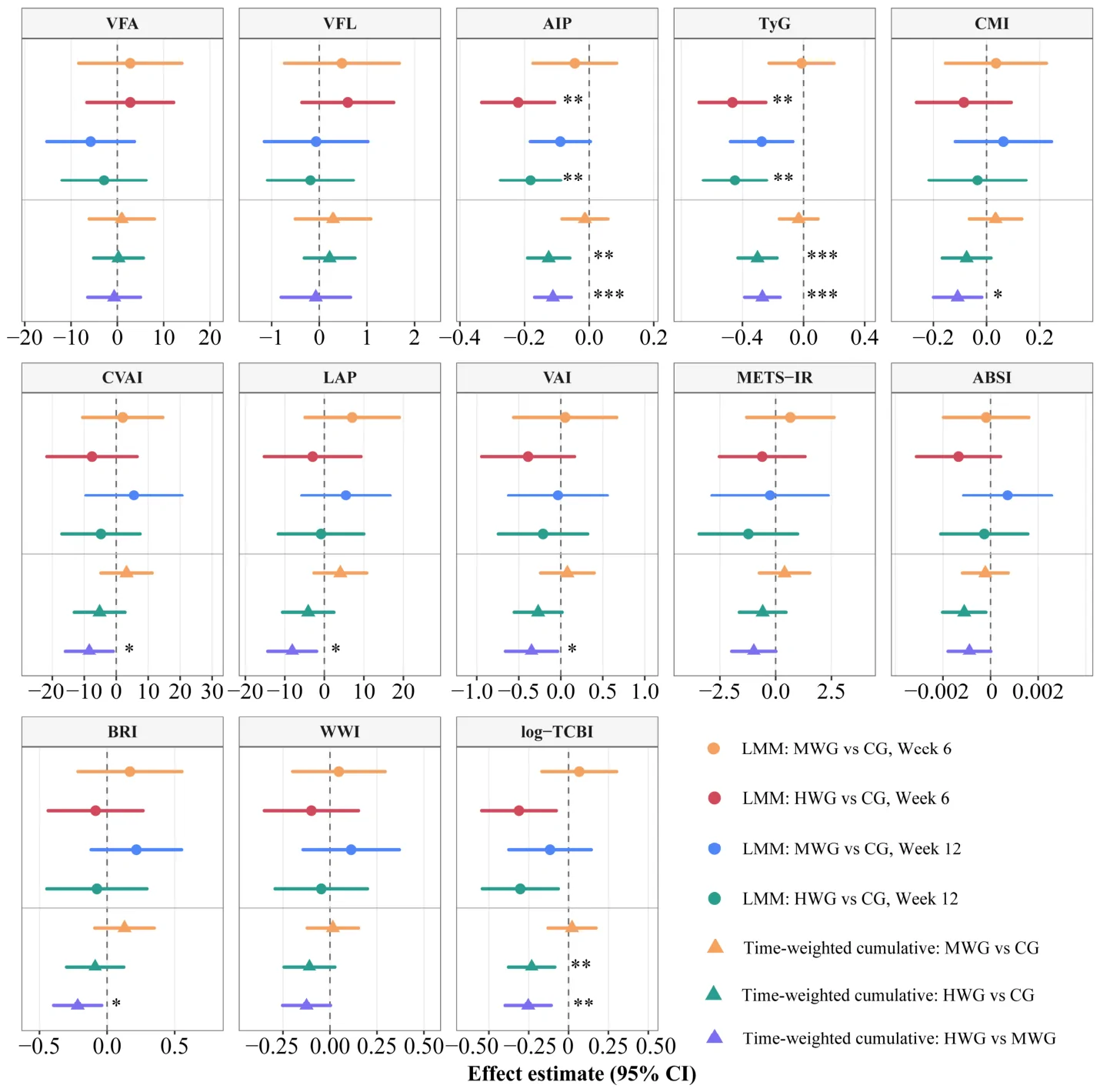
Longitudinal and time-weighted cumulative intervention effects on selected cardiometabolic phenotypes. The forest plots show intervention effect estimates for 13 selected cardiometabolic phenotypes. Circles represent group-by-time interaction estimates from longitudinal linear mixed-effects models at weeks 6 and 12, and triangles represent between-group differences in time-weighted cumulative change. Horizontal lines indicate 95% CIs, and the dashed vertical line denotes the null value. Asterisks indicate significance after FDR correction (* *p* < 0.05, ** *p* < 0.01, *** *p* < 0.001).

Linear mixed models at weeks 6 and 12 showed longitudinal differences across both directly measured and derived cardiometabolic outcomes. The broader longitudinal pattern involved AIP, TyG, conventional lipid components, and related cardiometabolic phenotypes, with no FDR-significant interaction effect for MWG versus CG across the extended phenotype panel (Table S5, Figure 2).

Time-weighted cumulative analyses showed effects across triglyceride-related measures and glucose–triglyceride coupling phenotypes. Compared with CG, HWG showed lower cumulative TG (β = −0.220, 95% CI −0.397 to −0.043; FDR-adjusted *p* = 0.047), AIP (β = −0.125, 95% CI −0.191 to −0.060; FDR-adjusted *p* = 0.002), and TyG (β = −0.301, 95% CI −0.429 to −0.173; FDR-adjusted *p* < 0.001). HDL-C showed a positive cumulative difference, whereas the TC contrast reflected the larger decline in CG. No FDR-significant cumulative effect was observed for MWG versus CG across the remaining phenotype panel. In the HWG versus MWG comparison, additional differences involved AIP, TyG, TG/HDL-C, and selected adiposity-related composite phenotypes (Table S5, Figure 2).

Sensitivity analyses restricted to the 134 participants who completed all three scheduled assessments yielded effect estimates generally similar in direction to those from the main analyses, with largely overlapping 95% CIs and no material change in the overall pattern of findings (Table S7).

### 3.2. NHANES Population Analysis Results

The NHANES analysis included 30,769 adults, of whom 12,169 met the definition of prediabetes (Table S8). When grouped by whole grain as a proportion of total grain intake, both the main sample and the prediabetes subgroup showed a clear grain-quality gradient: higher tertiles had higher whole-grain intake density, higher whole-grain/refined-grain ratio, and higher dietary fiber intake density, as well as lower refined-grain intake density. Distributions of demographic characteristics, lifestyle factors, dietary exposures, and metabolic indicators across groups are shown in Tables S9 and S10. Analyses of fasting indicator-related outcomes were based on 14,718 and 7739 participants with fasting subsample weights, respectively (Table S11).

Among 30,769 NHANES adults, grain-quality-related exposures showed consistent directions of association with body shape, abdominal adiposity, and glucose–lipid composite indicators. Higher whole-grain intake density was associated with lower waist circumference, BMI, BRI, WWI, METS-IR, CVAI, LAP, log(TCBI), and TyG-related body shape composite indicators; 20 outcomes were significant after FDR adjustment, all in the negative direction. Key results included waist circumference (standardized β −0.055, 95% CI −0.071 to −0.040; FDR-adjusted *p* < 0.001), BMI (−0.049, −0.065 to −0.033; FDR-adjusted *p* < 0.001), and TyG-WHtR (−0.068, −0.093 to −0.044; FDR-adjusted *p* < 0.001). Whole grain as a proportion of total grain intake showed broader associations, with 27 outcomes significant after FDR adjustment, including 26 negative associations mainly involving abdominal adiposity, body shape distribution, TyG-related indicators, METS-IR, CVAI, and LAP; HDL-C showed a positive association. Standardized β values for TyG-WHtR and HDL-C associated with whole-grain proportion were −0.088 (95% CI −0.112 to −0.065) and 0.035 (0.018 to 0.052), respectively, both with FDR-adjusted *p* < 0.001. Results for winsorized whole-grain intake density were similar to those for the original density. In contrast, total-grain intake density showed a narrower association pattern, with only 13 outcomes significant after FDR adjustment.

**Figure 3:**
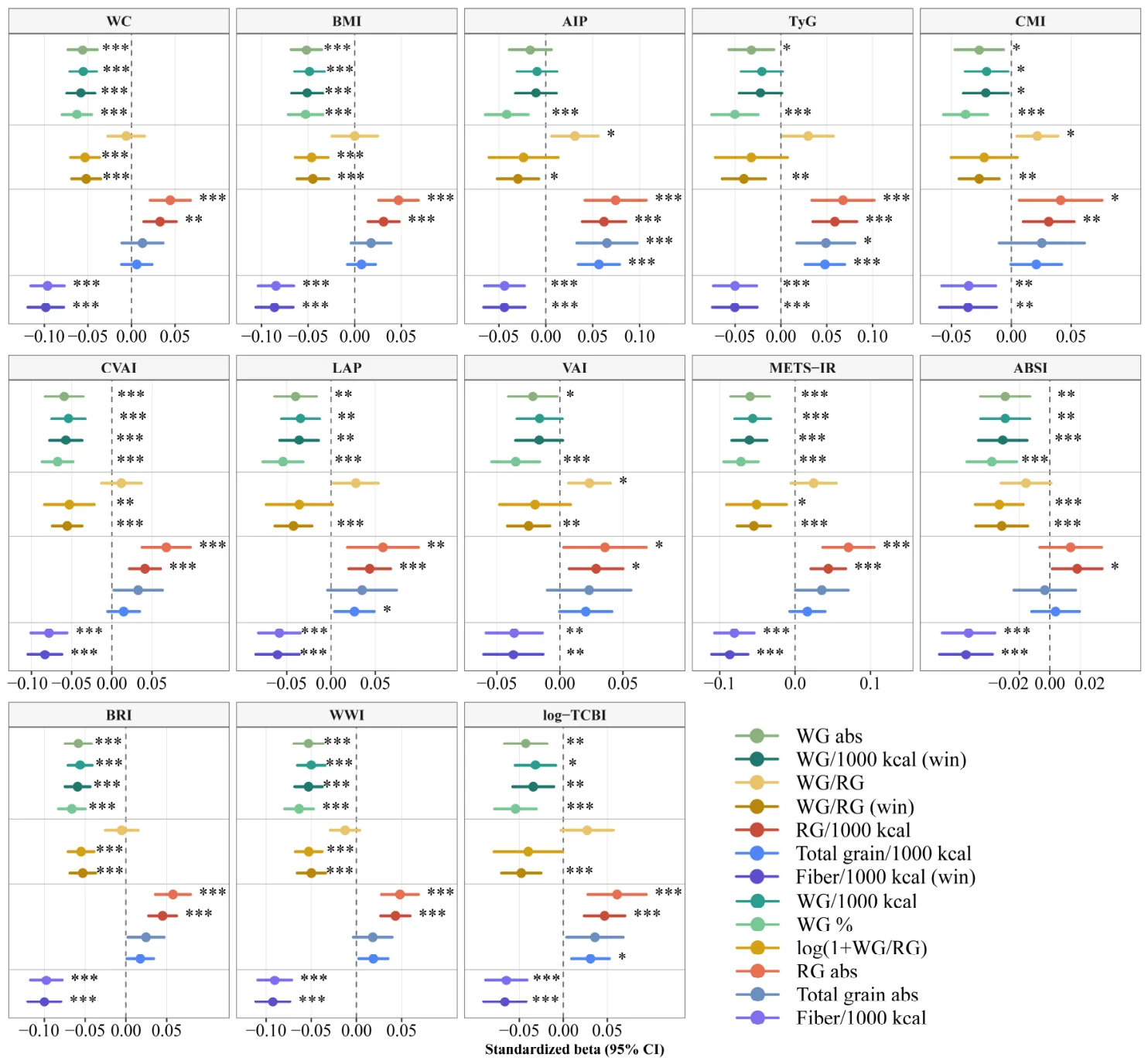
Dietary grain quality associations with selected cardiometabolic phenotypes in NHANES adults. This figure shows selected urvey-weighted linear regression results for dietary grain exposures and cardiometabolic phenotypes among 30,769 adults without diabetes in NHANES 2005–2006 to 2017–March 2020 prepandemic cycles. Point estimates are standardized regression coefficients, representing the difference in standardized outcome level per 1-SD increase in exposure; horizontal lines indicate 95% confidence intervals. Models incorporated NHANES complex sampling design weights, strata, and primary sampling units. Asterisks indicate statistical significance after false discovery rate correction (* *p* < 0.05, ** *p* < 0.01, and *** *p* < 0.001).

Refined-grain intake density showed directions opposite to those of whole-grain-related exposures. Higher refined-grain intake density was associated with higher AIP, TyG-WHtR, and most body shape, abdominal adiposity, and composite metabolic indicators, as well as with lower HDL-C and TC; 28 outcomes were significant after FDR adjustment. Key results included AIP (standardized β 0.062, 95% CI 0.039 to 0.085; FDR-adjusted *p* < 0.001), TyG-WHtR (0.062, 0.040 to 0.084; FDR-adjusted *p* < 0.001), and HDL-C (−0.076, −0.091 to −0.061; FDR-adjusted *p* < 0.001). Dietary fiber intake density was inversely associated with most body shape, abdominal adiposity, and composite metabolic indicators, whereas HDL-C showed a positive association. After winsorization, the same directional pattern was retained, with FDR-significant associations across the evaluated phenotypes (Table S12, Figure 3).

Among 12,169 adults with prediabetes, the association directions were consistent with the overall NHANES adult sample, but significant results for whole-grain intake density were concentrated mainly in waist circumference, BMI, and TyG-related body shape composite indicators. Whole-grain intake density was significantly associated with 13 outcomes after FDR adjustment, all in the negative direction, including waist circumference (standardized β −0.075, 95% CI −0.100 to −0.050; FDR-adjusted *p* < 0.001), BMI (−0.070, −0.093 to −0.048; FDR-adjusted *p* < 0.001), and TyG-WHtR (−0.074, −0.105 to −0.043; FDR-adjusted *p* < 0.001). Whole grain, as a proportion of total grain intake, remained significantly associated with 27 outcomes after FDR adjustment, of which 26 were negative and HDL-C was positive. Refined-grain intake density was significantly associated with 28 outcomes after FDR adjustment, mainly as positive associations with AIP, TyG-WHtR, and body shape composite indicators, and negative associations with HDL-C, TC, and LDL-C; standardized β values for AIP, TyG-WHtR, and HDL-C were 0.064 (95% CI 0.032 to 0.096), 0.082 (0.052 to 0.112), and −0.059 (−0.085 to −0.033), respectively (Table S13, Figure S2).

RCS analysis showed that grain-quality exposures were directionally consistent with the indicator clusters observed in linear models. Among NHANES adults, absolute whole-grain intake, whole-grain intake density, and the corresponding winsorized form were mainly associated with negative curves for the body shape–abdominal adiposity–TyG composite indicator cluster, involving waist circumference, BMI, BRI, WWI, TyG-WC, TyG-BMI, TyG-WHtR, TyG-WWI, and TyG-related visceral adiposity indicators; METS-IR, CVAI, LAP, and log(TCBI) also decreased with increasing whole-grain exposure. Whole grain, as a proportion of total grain intake, showed a broader range of curve associations, with negative relationships covering the above body shape, abdominal adiposity, TyG-related composite indicators, and visceral adiposity-related indicators, and a positive curve for HDL-C. Whole-grain/refined-grain ratio exposures, log-transformed ratio exposures, and winsorized ratio exposures showed the same directions for these indicator clusters, with some nonlinear associations also observed.

**Figure 4:**
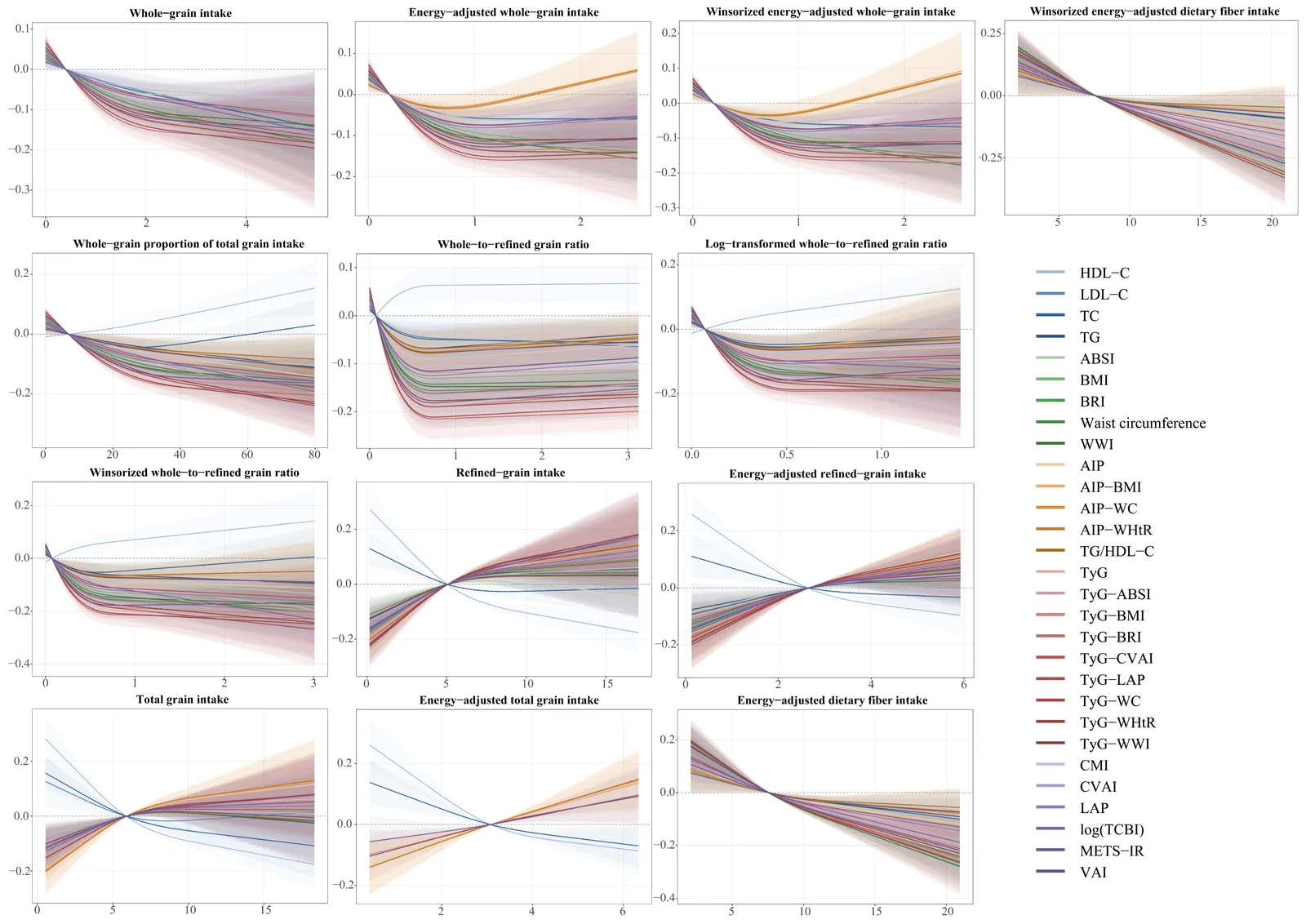
Nonlinear dietary grain associations with NHANES metabolic phenotypes. This figure shows nonlinear cross-sectional associations between dietary grain exposures and cardiometabolic phenotypes among NHANES adults. Each panel corresponds to one dietary grain exposure; curves represent survey-weighted restricted cubic spline model estimates of exposure-outcome relationships, and shaded regions indicate 95% confidence intervals.

The directions of RCS curves for refined-grain exposures were opposite to those for whole-grain exposures. Absolute refined-grain intake and refined-grain intake density showed positive curves with the body shape–abdominal adiposity–TyG composite indicator cluster, AIP, METS-IR, CVAI, and LAP, and negative curves with HDL-C and TC. Absolute total-grain intake and total-grain intake density showed fewer curve associations, and their directions were less consistent than those of grain-quality indicators; their main positive curves were concentrated in selected body shape, abdominal adiposity, and composite metabolic indicators. Dietary fiber intake density and its winsorized form showed directions consistent with whole-grain exposures, with negative curves across multiple outcomes, mainly involving body shape, abdominal adiposity, TyG-related composite indicators, METS-IR, CVAI, and LAP (Table S14, Figure 4).

Among adults with prediabetes, the RCS curves retained the same exposure-indicator cluster structure. Negative curves for whole-grain intake density and its winsorized form were concentrated mainly in the body shape–abdominal adiposity–TyG composite indicator cluster; whole grain as a proportion of total grain intake and whole-grain/refined-grain ratio-related exposure showed a broader range of negative curves, covering abdominal adiposity, body shape distribution, TyG-related indicators, and visceral adiposity-related composite indicators. Absolute refined-grain intake and refined-grain intake density were still mainly associated with positive curves for body shape, abdominal adiposity, AIP, TyG, and TyG-related composite indicators, and negative curves for HDL-C, TC, or LDL-C. Total-grain exposure showed fewer curve associations; dietary fiber intake density and its winsorized form showed negative curves mainly corresponding to body shape and glucose–lipid composite risk indicators (Table S15, Figure S3).

### 3.3. Country-Level Contextual Analysis Results

The country-level analysis included 163 countries across the three time points of 2010, 2015, and 2018, yielding 489 country-year observations. In 2010, 2015, and 2018, median whole-grain intake was 36.3, 32.7, and 35.3 g/day, respectively; median high-FPG-attributable diabetes DALY rates were 1068.9, 1109.3, and 1126.1; and median cereal supply was 150.0, 152.5, and 149.8 kg/capita/year. In 2018, whole-grain intake ranged from 0.05 to 194.85 g/day, high-FPG-attributable diabetes DALY rates ranged from 329.2 to 7455.8, and cereal supply ranged from 42.0 to 337.1 kg/capita/year (Tables S16 and S17A, Figure S4).

Using empirical medians from pooled country-year observations in 2010, 2015, and 2018 as descriptive cut-points, 47 countries (28.8%) in 2018 had both lower whole-grain intake and higher high-FPG-attributable diabetes burden. Of these, 23 had cereal supply above the pooled cut-point and 24 below it. A further 39 countries (23.9%) combined higher whole-grain intake with higher diabetes burden, whereas 44 countries (27.0%) combined higher whole-grain intake with lower burden. Detailed distributions of these combinations across years and SDI strata are provided in Tables S18 and S19 and Figure S5.

## 4. Discussion

The randomized intervention showed the most consistent changes in triglyceride-related lipid composition and glucose–triglyceride coupling. The week-12 between-group differences in FPG did not remain significant after FDR correction. The HWG showed lower cumulative TG than the CG, and HDL-C showed favorable between-group changes at both follow-up visits. TC followed a different temporal trajectory, with part of the between-group contrast arising from a larger decline in CG; LDL-C did not show a stable FDR-significant difference. AIP and TyG showed the most concentrated patterns across the extended phenotype analyses.

Correlation analysis showed that multiple significant indicators shared core components, including TG, HDL-C, waist circumference, BMI, or TyG/AIP. AIP-related indicators primarily reflected the TG–HDL-C axis and atherogenic dyslipidemia; TyG-related indicators reflected triglyceride–glucose coupling and insulin resistance-related phenotypes; and VAI, CVAI, LAP, BRI, ABSI, and related indicators more strongly characterized abdominal adiposity and visceral adiposity-related risk. These concentrated changes in composite indicators are consistent with correlated shifts in shared metabolic components and should be interpreted in light of the mathematical dependence among these indices.

Previous whole-grain intervention trials have reported heterogeneous metabolic responses according to food matrix, replacement target, energy context, and participant phenotype. Giacco et al. observed lower postprandial insulin and triglyceride responses after a whole-grain cereal diet in adults with metabolic syndrome [46], whereas Cooper et al. reported changes in fasting cholesterol with more modest glycemic effects [47]. Tighe et al. primarily observed blood pressure reductions in healthy middle-aged adults [48], and the RyeWeight trial found greater reductions in body weight and body fat with a high-fiber rye diet under energy restriction [49]. Across these trials, responsive outcomes frequently involved triglyceride handling, insulin-related physiology, or adiposity, consistent with the concentration of signals observed in the present extended phenotype analysis [46,49,50].

The metabolic response to whole-grain foods is shaped by grain structure, processing, and the composition of the food matrix. More intact cereal structures can alter starch accessibility and postprandial glucose handling, while food microstructure influences gastric processing and digestive kinetics [51,52]. Dietary fiber can further modify intestinal viscosity, fermentation, short-chain fatty acid production, gastrointestinal signaling, and lipid metabolism [53,54]. The intervention used both rice- and wheat-based whole-grain staples, and the observed metabolic changes are consistent with combined changes in grain structure, dietary fiber, and the overall staple-food matrix.

NHANES showed cross-sectional metabolic patterns associated with habitual grain quality in free-living adults. Higher whole-grain density, whole-grain proportion, and grain-quality ratios were associated with lower waist circumference, BMI, METS-IR, CVAI, LAP, and several TyG-related phenotypes. Refined-grain density generally showed the opposite association pattern. Associations for total-grain intake were more limited, indicating that the relative contribution of whole and refined grains captured more of the observed variation in cardiometabolic phenotypes.

Current dietary guidance across multiple settings emphasizes the quality and composition of staple carbohydrate sources. WHO guidance prioritizes whole grains, vegetables, fruits, and pulses as carbohydrate sources, while Chinese, US, UK, Australian, and Canadian recommendations similarly encourage whole-grain or higher-fiber grain choices [55,56,57,58,59,60]. In staple-based diets, these recommendations translate naturally into changes in the proportion and form of grain foods consumed within habitual meals, which is the dietary context represented by the standardized rice- and wheat-based products used in this trial.

The NHANES prediabetes subgroup provided a population context closely aligned with the glycemic-risk profile of the randomized cohort. Prediabetes frequently coexists with dyslipidemia and other cardiovascular risk factors [61], and lifestyle intervention can reduce progression to diabetes while improving cardiometabolic risk factors [62]. In the present analysis, grain-quality associations in this subgroup were concentrated in waist circumference, BMI, and TyG-related adiposity phenotypes, corresponding to the abdominal adiposity and insulin resistance clustering that commonly precedes overt diabetes.

Longitudinal cohort studies further place these intermediate phenotypes within the longer cardiometabolic risk continuum. Higher whole-grain intake has been associated with lower incident type 2 diabetes risk and more favorable trajectories of waist circumference and selected metabolic factors, while refined-grain intake has shown less favorable associations [21,63,64]. Prospective studies in China and Korea have likewise linked coarse- or whole-grain intake with lower risks of diabetes, ischemic stroke, or metabolic syndrome [65,66]. The present trial captures an earlier stage of this continuum by characterizing short-term metabolic responses before disease incidence can be evaluated.

Country-level analyses further showed that whole-grain intake, high-FPG-attributable diabetes burden, and cereal supply occur in complex combinations across countries. Among the 47 countries with lower whole-grain intake and higher diabetes burden in 2018, nearly equal numbers had cereal supply above and below the pooled cut-point; substantial diabetes burden also occurred in a subset of countries with relatively higher whole-grain intake. Low whole-grain intake has established public health relevance [23]. At the population level, overall dietary patterns, obesity and related metabolic risk factors, population structure, socioeconomic conditions, and the stage of nutrition transition can all shape chronic disease burden [67,68]. Similar whole-grain intake levels or cereal supply conditions may therefore coexist with markedly different metabolic disease profiles.

Cereal supply helps locate these country-level patterns within the food system because national availability represents only an upstream condition for what ultimately enters the diet. Grains pass through milling and other forms of processing, product formulation, distribution, retail, pricing, and household purchasing before reaching consumers, and each stage can alter both the amount and the form of whole grains that remain available for consumption. Countries with ample cereal supply may therefore still have low whole-grain intake when refining predominates, whole-grain products are less available or less affordable, or prevailing food environments and habitual food choices favor refined staples. Evidence linking food supply, food prices, and food environments to diet quality, together with persistent affordability constraints on healthy diets, supports this interpretation [69,70]. The country combinations observed here are consistent with such a supply-to-consumption pathway and help explain why similar levels of cereal availability can coexist with markedly different whole-grain intakes and diabetes burdens. The present supply indicator does not distinguish grain type, processing degree, product availability, price, or household access, leaving these intermediate stages unresolved. Product-level supply and consumption data would allow future analyses to identify more precisely where along this pathway barriers to whole-grain intake arise.

Several considerations define the interpretation of these findings. The randomized intervention lasted 12 weeks and was conducted in adults at high risk of type 2 diabetes, so the observed phenotype changes primarily characterize short-term metabolic response in this population. The intervention was delivered using standardized steamed-bun and rice-based staple preparations with formulation-specific composition, so the treatment effect reflects the implemented whole-grain replacement strategy rather than a single formulation. Adherence was actively managed through daily records, electronic scales, biweekly record collection, remaining food checks, and regular follow-up; the retained analytic archive did not provide a uniform participant-level field from which a standardized adherence percentage could be reconstructed for all participants. The control group maintained its habitual diet and received no isocaloric refined-grain comparator. Complete-case sensitivity estimates were broadly consistent with the main analyses. NHANES relied on cross-sectional dietary recalls and remains subject to within-person dietary variation and residual confounding, while the country-level analysis describes ecological combinations of whole-grain intake, diabetes burden, and cereal supply. The country-level typology used empirical medians from pooled country-year observations as descriptive cut-points, and classifications may vary with the thresholds selected. Longer interventions and studies conducted in diverse staple-food environments will be valuable for assessing the persistence and broader applicability of these metabolic responses.

## 5. Conclusions

In this secondary exploratory analysis of a randomized dietary trial, short-term cardiometabolic changes after whole-grain staple replacement were concentrated in triglyceride-related lipid composition and glucose–triglyceride coupling. NHANES showed cross-sectional associations between habitual grain quality and cardiometabolic phenotypes, and the country-level analysis described substantial heterogeneity in whole-grain intake, high-FPG-attributable diabetes burden, and cereal supply context. These findings support further evaluation of staple-based whole-grain replacement over longer intervention periods and in diverse habitual dietary settings.

## Data Availability

The NHANES data analyzed in this study are publicly available from the National Center for Health Statistics. Country-level whole-grain intake, high-fasting-plasma-glucose-attributable diabetes burden, and cereal supply data were obtained from the Global Dietary Database, the Global Burden of Disease Study Results Tool, and FAOSTAT Food Balance Sheets, respectively. Deidentified participant-level data from the randomized trial, the accompanying data dictionary, and analytic code are available from the corresponding author upon reasonable request, subject to applicable ethical and data protection requirements. Supporting figures and tables are provided in the Supplementary Materials.

## Supplementary Materials

The following supporting information can be downloaded at https://www.mdpi.com/article/10.3390/nu18172758/s1: Figure S1: Study flow diagram for the randomized intervention, NHANES cross-sectional analysis, and country-level contextual analysis; Figure S2: Grain-quality associations with cardiometabolic phenotypes in adults with prediabetes; Figure S3: Nonlinear grain-quality associations with cardiometabolic phenotypes in adults with prediabetes; Figure S4: Global distribution of country-level whole-grain intake, high-FPG-attributable diabetes burden, and cereal supply context; Figure S5: Country-level descriptive typology based on whole-grain intake, high-FPG-attributable diabetes burden, and cereal supply context; Table S1: Composition and nutritional characteristics of the standardized whole-grain staple products; Table S2: Baseline characteristics of RCT participants by randomized group; Table S3: Grain intake and whole-grain exposure before and during the randomized intervention; Table S4: Changes from baseline and time-weighted cumulative changes in RCT metabolic indicators; Table S5: Linear mixed-model interaction estimates and time-weighted cumulative effect estimates for RCT metabolic indicators; Table S6: Correlation matrix for time-weighted cumulative changes in RCT metabolic indicators; Table S7: Sensitivity estimates for RCT metabolic indicators; Table S8: NHANES sample structure, tertile cutoffs, dietary exposure distributions, and outcome definitions; Table S9: Characteristics of the NHANES main analytic sample by tertiles of whole grain as a proportion of total grain intake; Table S10: Characteristics of NHANES participants with prediabetes by tertiles of whole grain as a proportion of total grain intake; Table S11: Outcome-specific complete-case sample sizes for NHANES survey-weighted models; Table S12: Survey-weighted linear associations between grain-quality exposures and metabolic outcomes in the NHANES main analytic sample; Table S13: Survey-weighted linear associations between grain-quality exposures and metabolic outcomes in NHANES participants with prediabetes; Table S14: Restricted cubic spline associations between grain-quality exposures and metabolic outcomes in the NHANES main analytic sample; Table S15: Restricted cubic spline associations between grain-quality exposures and metabolic outcomes in NHANES participants with prediabetes; Table S16: Country-year whole-grain intake, high-FPG-attributable diabetes burden, cereal supply, and descriptive typology, 2010, 2015, and 2018; Table S17: Overall and regional distributions of whole-grain intake, high-FPG-attributable diabetes burden, and cereal supply; Table S18: Definitions, yearly composition, and regional distribution of the country-level descriptive typology; Table S19: Characteristics of the 2018 descriptive typology and distributions by SDI category.

## Abbreviations

The following abbreviations are used in this manuscript:

Abbreviation	Full term
ABSI	A body shape index
ADA	American Diabetes Association
AIC	Akaike information criterion
AIP	Atherogenic index of plasma
BMI	Body mass index
BRI	Body roundness index
CG	Control group
CI	Confidence interval
CMI	Cardiometabolic index
CVAI	Chinese visceral adiposity index
DALY	Disability-adjusted life-year
FAOSTAT	Food and Agriculture Organization Corporate Statistical Database
FDR	False discovery rate
FPED	Food Patterns Equivalents Database
FPG	Fasting plasma glucose
GBD	Global Burden of Disease Study
GDD	Global Dietary Database
HDL-C	High-density lipoprotein cholesterol
HbA1c	Hemoglobin A1c
HWG	High-dose whole-grain group
IQR	Interquartile range
LAP	Lipid accumulation product
LDL-C	Low-density lipoprotein cholesterol
LMM	Linear mixed-effects model
METS-IR	Metabolic score for insulin resistance
MWG	Medium-dose whole-grain group
NCHS	National Center for Health Statistics
NHANES	National Health and Nutrition Examination Survey
PSU	Primary sampling unit
RCS	Restricted cubic spline
RCT	Randomized controlled trial
SD	Standard deviation
SDI	Socio-demographic Index
TC	Total cholesterol
TCBI	Triglyceride–cholesterol–body weight index
TG	Triglycerides
TyG	Triglyceride–glucose index
VAI	Visceral adiposity index
WC	Waist circumference
WHO	World Health Organization
WHtR	Waist-to-height ratio
WWI	Weight-adjusted waist index
