# Supplementary material for: Whole-Grain Staple Replacement and Cardiometabolic Phenotypes in Adults at High Risk of Type 2 Diabetes: An Extended Randomized-Trial Analysis with NHANES and Country-Level Context
Source: Nutrients. 2026 Aug 23;18(17):2758. doi: 10.3390/nu18172758 (PMC13567143; doi:10.3390/nu18172758)
Supplement: Supplementary file 1 [file nutrients-18-02758-s001.zip › CONSORT_2025_Checklist.pdf]

CONSORT 2025 checklist of information to include when reporting a randomised trial*

Section / Topic	No	CONSORT 2025 checklist item description	Reported in final manuscript
Title and abstract			
Title and structured abstract	1a	Identification as a randomised trial	p. 1, manuscript title (identifies the report as a randomized-trial analysis).
	1b	Structured summary of the trial design, methods, results, and conclusions	pp. 1 – 2, structured Abstract.
Open science			
Trial registration	2	Name of trial registry, identifying number (with URL) and date of registration	Abstract, Trial registration; Section 2.1.1.1, Study Design and Participants (Chinese Clinical Trial Registry, ChiCTR2300072952; registered 28 June 2023).
Protocol and statistical analysis plan	3	Where the trial protocol and statistical analysis plan can be accessed	Trial registration (ChiCTR2300072952) and Sections 2.1 – 2.5, which report the trial design, intervention, phenotype framework, and statistical methods used for the present analysis.
Data sharing	4	Where and how the individual de-identified participant data (including data dictionary), statistical code and any other materials can be accessed	Data Availability Statement. Deidentified participant-level trial data, the accompanying data dictionary, and analytic code are available from the corresponding author upon reasonable request, subject to applicable ethical and data-protection requirements.
Funding and conflicts of interest	5a	Sources of funding and other support (e.g., supply of drugs), and role of funders in the design, conduct, analysis and reporting of the trial	Funding statement; the roles of the funders are described in the Conflicts of Interest statement.
	5b	Financial and other conflicts of interest of the manuscript authors	Conflicts of Interest statement.
Introduction			
Background and rationale	6	Scientific background and rationale	Section 1, Introduction.
Objectives	7	Specific objectives related to benefits and harms	Final paragraph of Section 1, Introduction; safety monitoring is described in Section 2.1.1.3 and summarized in Section 3.1.
Methods			
Patient and public involvement	8	Details of patient or public involvement in the design, conduct and reporting of the trial	Not applicable to the present secondary exploratory analysis.
Trial design	9	Description of trial design including type of trial (e.g., parallel group, crossover), allocation ratio, and framework (e.g., superiority, equivalence, non-inferiority, exploratory)	Section 2.1.1, Randomized Controlled Dietary Intervention. The report identifies a 12-week, three-arm, parallel randomized dietary trial and the present secondary exploratory analysis.
Changes to trial protocol	10	Important changes to the trial after it commenced including any outcomes or analyses that were not prespecified, with reason	Sections 2.1.1, 2.2, and 2.5. The expanded cardiometabolic phenotype analyses are explicitly identified and described as secondary exploratory analyses.
Trial setting	11	Settings (e.g., community, hospital) and locations (e.g., countries, sites) where the trial was conducted	Section 2.1.1.1, Study Design and Participants; Institutional Review Board Statement.
Eligibility criteria	12a	Eligibility criteria for participants	Section 2.1.1.1, Study Design and Participants.
	12b	If applicable, eligibility criteria for sites and for individuals delivering the interventions (e.g., surgeons, physiotherapists)	Not applicable.
Intervention and comparator	13	Intervention and comparator with sufficient details to allow replication. If relevant, where additional materials describing the intervention and comparator (e.g., intervention manual) can be accessed	Sections 2.1.1.2 – 2.1.1.3, Randomization and Dietary Intervention and Dietary and Lifestyle Assessment; Table S1 provides product formulation and available nutritional characteristics.
Outcomes	14	Pre-specified primary and secondary outcomes, including the specific measurement variable (e.g., systolic blood pressure), analysis metric (e.g., change from baseline, final value, time to event), method of aggregation (e.g., median, proportion), and time point for each outcome	Sections 2.1.1.1, 2.2, 2.5, and 3.1. The present report is explicitly identified as a secondary exploratory analysis. FPG is reported as a directly measured glycemic indicator, while the lipid, anthropometric, body-composition, and derived cardiometabolic phenotype analyses constitute the extended exploratory focus of the present report.

Harms	15	How harms were defined and assessed (e.g., systematically, non-systematically)	Section 2.1.1.3 and Section 3.1. Medication use and major health events were recorded during follow-up; no serious adverse events clearly related to the study foods were reported.
Sample size	16a	How sample size was determined, including all assumptions supporting the sample size calculation	Section 2.1.1.2. The present secondary analysis included 144 participants from the registered randomized cohort, with 48 participants in each randomized group.
	16b	Explanation of any interim analyses and stopping guidelines	Not applicable.
Randomisation:			
Sequence generation	17a	Who generated the random allocation sequence and the method used	Section 2.1.1.2. The random allocation sequence was generated by an independent statistician using a computer-generated procedure.
	17b	Type of randomisation and details of any restriction (e.g., stratification, blocking and block size)	Section 2.1.1.2. Block randomization with a 1:1:1 allocation ratio is described.
Allocation concealment mechanism	18	Mechanism used to implement the random allocation sequence (e.g., central computer/telephone; sequentially numbered, opaque, sealed containers), describing any steps to conceal the sequence until interventions were assigned	Section 2.1.1.2. Allocation concealment used sequentially numbered, opaque, sealed envelopes.
Implementation	19	Whether the personnel who enrolled and those who assigned participants to the interventions had access to the random allocation sequence	Section 2.1.1.2. Sequence generation was performed by an independent statistician and allocation concealment was maintained using sequentially numbered, opaque, sealed envelopes.
Blinding	20a	Who was blinded after assignment to interventions (e.g., participants, care providers, outcome assessors, data analysts)	Section 2.1.1.2. Participants and field staff could not be blinded because of the dietary intervention; laboratory personnel and statistical analysts remained blinded to group assignment until completion of the main analyses.
	20b	If blinded, how blinding was achieved and description of the similarity of interventions	Section 2.1.1.2, Randomization and Dietary Intervention.
Statistical methods	21a	Statistical methods used to compare groups for primary and secondary outcomes, including harms	Section 2.5, Statistical Analysis; safety outcomes are summarized descriptively in Section 3.1.
	21b	Definition of who is included in each analysis (e.g., all randomised participants), and in which group	Sections 2.1.1.2 and 2.5.1. Participants were analyzed according to their original randomized assignments, and all available repeated observations contributed to the main longitudinal models.
	21c	How missing data were handled in the analysis	Section 2.5.1. Likelihood-based longitudinal models used all available repeated observations; sensitivity analyses were repeated in the 134 participants who completed all three scheduled assessments.
	21d	Methods for any additional analyses (e.g., subgroup and sensitivity analyses), distinguishing prespecified from post-hoc	Section 2.5.1. Three-visit completer sensitivity analyses used the same longitudinal and time-weighted cumulative model definitions and FDR correction structures as the main phenotype analyses.
Results			
Participant flow, including flow diagram	22a	For each group, the numbers of participants who were randomly assigned, received intended intervention, and were analysed for the primary outcome	Section 3.1 and Figure S1. The RCT analysis set comprised 144 participants, with 48 participants in each randomized group; participants were analyzed according to their original randomized assignments.
	22b	For each group, losses and exclusions after randomisation, together with reasons	Figure S1. Follow-up completion is shown by randomized group: week 6, HWG n = 46, MWG n = 48, CG n = 45; week 12, HWG n = 43, MWG n = 47, CG n = 44. The three-visit completer cohort comprised 134 participants.
Recruitment	23a	Dates defining the periods of recruitment and follow-up for outcomes of benefits and harms	Sections 2.1.1 and 3.1. The intervention lasted 12 weeks, with scheduled assessments at baseline, week 6, and week 12.
	23b	If relevant, why the trial ended or was stopped	Not applicable.
Intervention and comparator delivery	24a	Intervention and comparator as they were actually administered (e.g., where appropriate, who delivered the intervention/comparator, how participants adhered, whether they were delivered as intended [fidelity])	Sections 2.1.1.2 – 2.1.1.3; Tables S1 and S3. Study-food delivery and monitoring procedures are described, and intervention-period grain exposure is summarized using target-based estimated total whole-grain exposure and refined-grain intake.
	24b	Concomitant care received during the trial for each group	Section 2.1.1.3. All three groups received the same healthy-diet and lifestyle education materials; the control group maintained its habitual dietary pattern.
Baseline data	25	A table showing baseline demographic and clinical characteristics for each group	Table S2.

Numbers analysed, outcomes and estimation	26	For each primary and secondary outcome, by group:<br>the number of participants included in the analysis<br>the number of participants with available data at the outcome time point<br>result for each group, and the estimated effect size and its precision (such as 95% confidence interval)<br>for binary outcomes, presentation of both absolute and relative effect size	Section 3.1; Tables S4 – S5. Visit-specific, longitudinal, and time-weighted cumulative results are reported with effect estimates, 95% confidence intervals, and multiplicity-adjusted P values; the three-visit completer sensitivity analysis is reported in Table S7.
Harms	27	All harms or unintended events in each group	Section 3.1. No serious adverse events clearly related to the study foods were reported during the 12-week follow-up.
Ancillary analyses	28	Any other analyses performed, including subgroup and sensitivity analyses, distinguishing pre-specified from post-hoc	Sections 2.5.1 and 3.1; Tables S5 – S7. Additional analyses include time-weighted cumulative effects, correlation structure, and the three-visit completer sensitivity analysis.
Discussion			
Interpretation	29	Interpretation consistent with results, balancing benefits and harms, and considering other relevant evidence	Section 4, Discussion.
Limitations	30	Trial limitations, addressing sources of potential bias, imprecision, generalisability, and, if relevant, multiplicity of analyses	Section 4, Discussion (limitations paragraph).

*We strongly recommend reading this statement in conjunction with the CONSORT 2025 Explanation and Elaboration and/or the CONSORT 2025 Expanded Checklist for important clarifications on all the items. We also recommend reading relevant CONSORT extensions. See www.consort-spirit.org.

Citation: Hopewell S, Chan AW, Collins GS, Hróbjartsson A, Moher D, Schulz KF, et al. CONSORT 2025 Statement: updated guideline for reporting randomised trials. BMJ. 2025; 388:e081123. https://dx.doi.org/10.1136/bmj-2024-081123.

© 2025 Hopewell et al. This is an Open Access article distributed under the terms of the Creative Commons Attribution License (https://creativecommons.org/licenses/by/4.0/), which permits unrestricted use, distribution, and reproduction in any medium, provided the original work is properly cited.
