# Supplementary material for: Whole-Grain Staple Replacement and Cardiometabolic Phenotypes in Adults at High Risk of Type 2 Diabetes: An Extended Randomized-Trial Analysis with NHANES and Country-Level Context
Source: Nutrients. 2026 Aug 23;18(17):2758. doi: 10.3390/nu18172758 (PMC13567143; doi:10.3390/nu18172758)
Supplement: Supplementary file 1 [file nutrients-18-02758-s001.zip › Supplementary_Figures.pdf]

Supplementary Figures

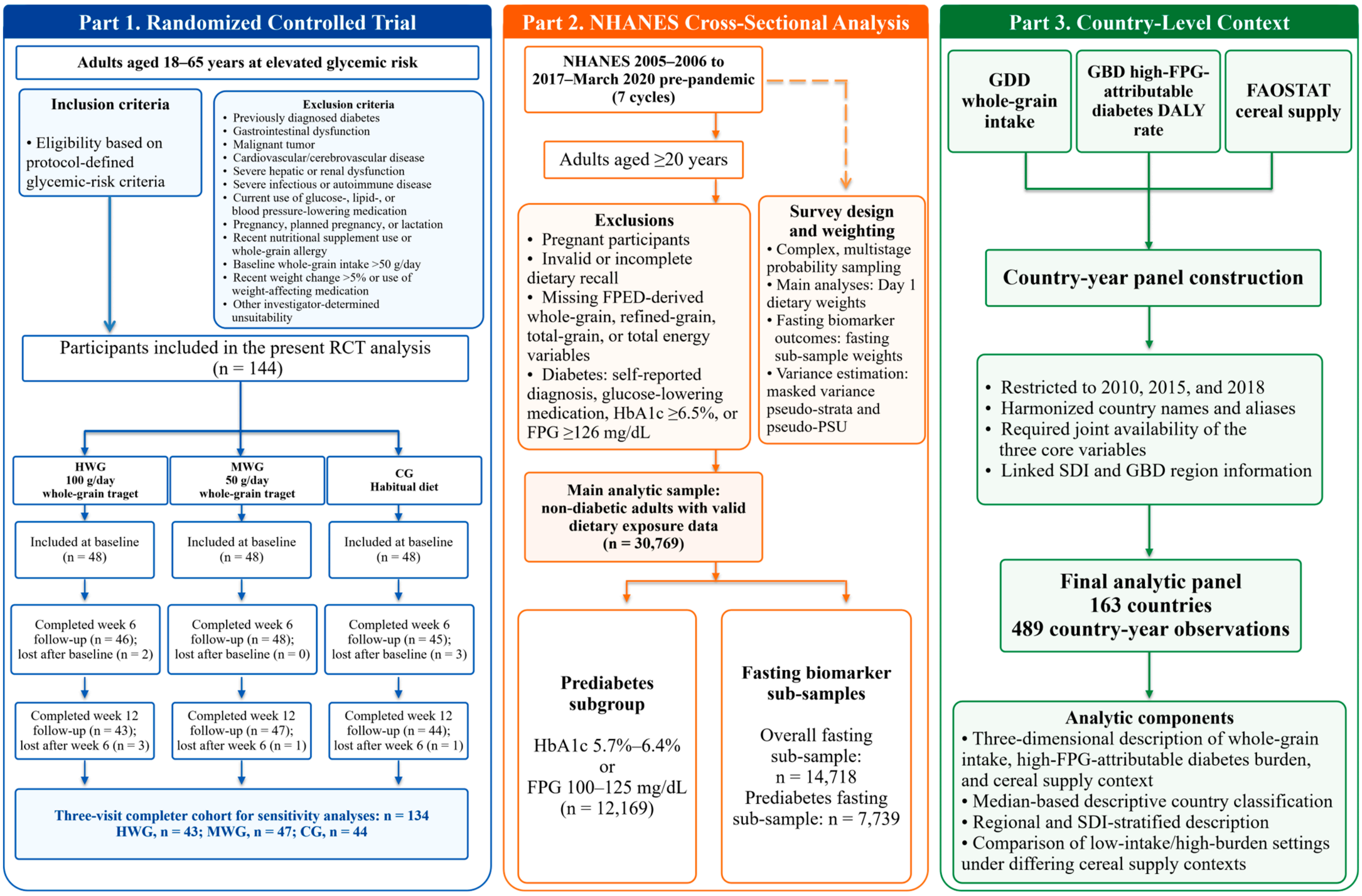

Figure S1. Study flow diagram for the randomized intervention, NHANES cross-sectional analysis, and country-level contextual analysis.

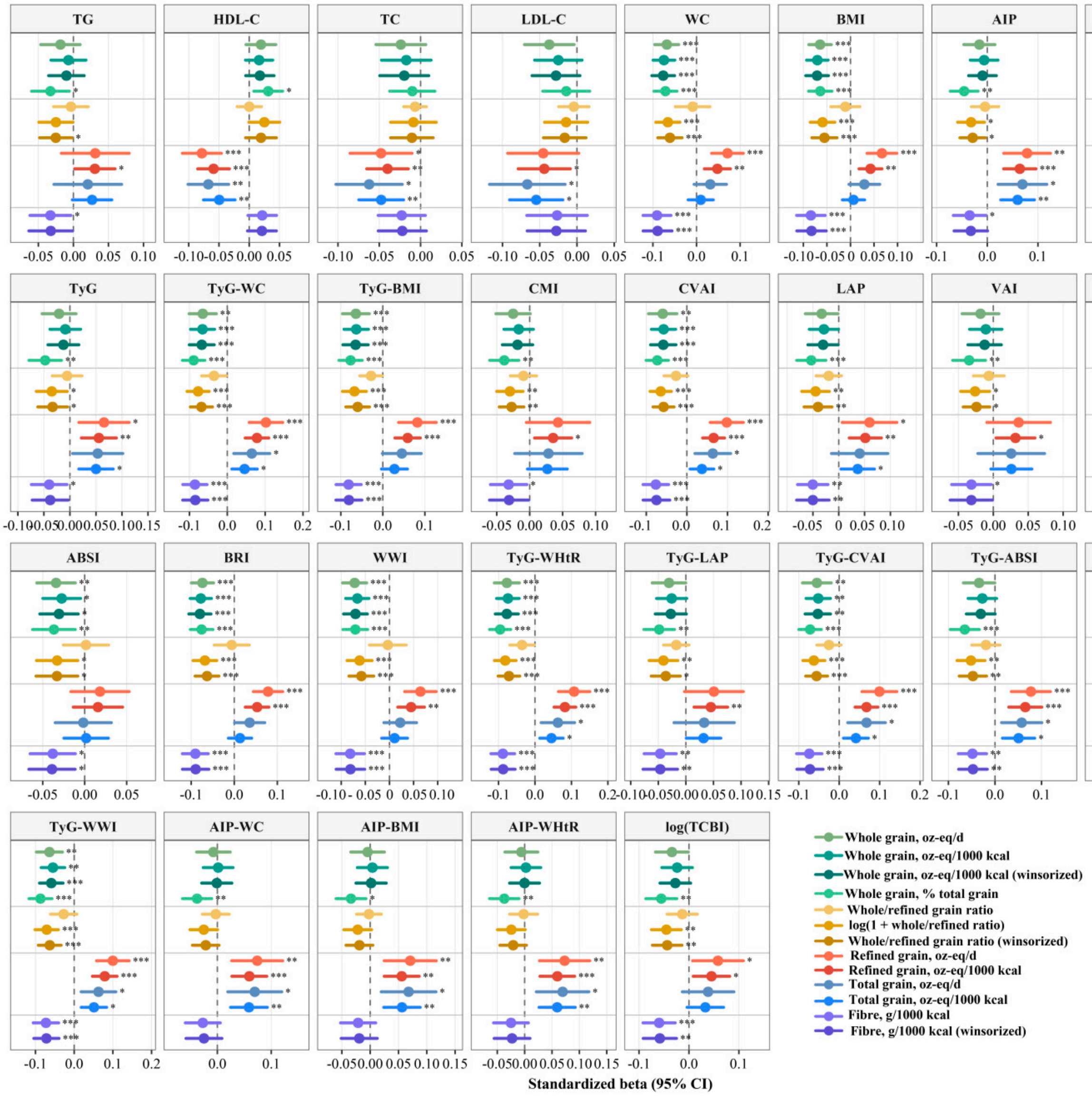

Figure S2. Grain-quality associations with cardiometabolic phenotypes in adults with prediabetes. This figure shows survey-weighted linear regression results for dietary grain exposures and available cardiometabolic phenotypes among 12,169 adults with prediabetes in NHANES 2005–2006 to the 2017–March 2020 prepandemic cycle. Point estimates are standardized regression coefficients, representing the difference in standardized outcome level per 1-SD increase in exposure; horizontal lines indicate 95% confidence intervals. Models were adjusted for age, sex, race or ethnicity, survey cycle, education level, family income-to-poverty ratio, smoking status, alcohol drinking status, physical activity status, and total energy intake, and incorporated NHANES complex sampling design weights, strata, and primary sampling units for variance estimation. Significance markers are based on FDR-adjusted P values. Abbreviations: ABSI, a body shape index; AIP, atherogenic index of plasma; BMI, body mass index; BRI, body roundness index; CMI, cardiometabolic index; CVAI, Chinese visceral adiposity index; FDR, false discovery rate; HDL-C, high-density lipoprotein cholesterol; LAP, lipid accumulation product; LDL-C, low-density lipoprotein cholesterol; METS-IR, metabolic score for insulin resistance; NHANES, National Health and Nutrition Examination Survey; PSU, primary sampling unit; TC, total cholesterol; TCBI, triglyceride-cholesterol-body weight index; TG, triglycerides; TyG, triglyceride-glucose index; VAI, visceral adiposity index; WC, waist circumference; WHtR, waist-to-height ratio; WWI, weight-adjusted waist index.

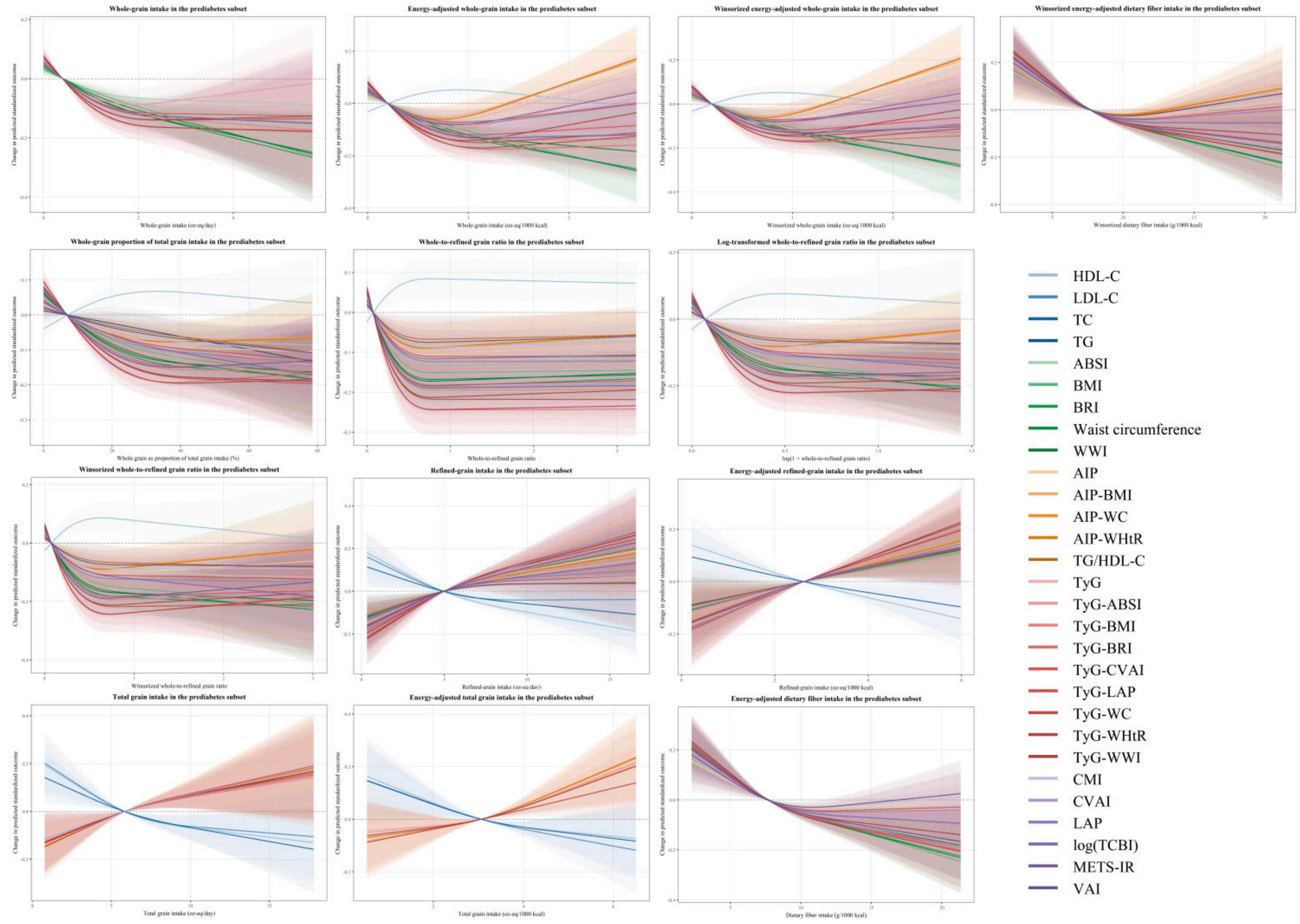

Figure S3. Nonlinear grain-quality associations with cardiometabolic phenotypes in adults with prediabetes. This figure shows nonlinear cross-sectional associations between dietary grain exposures and available cardiometabolic phenotypes among NHANES adults with prediabetes. Each panel corresponds to one dietary grain exposure; curves represent survey-weighted restricted cubic spline model estimates of exposure−outcome relationships, and shaded regions indicate 95% confidence intervals. The number of spline knots was selected from three, four, and five candidate values according to the Akaike information criterion, and curves used the median of the corresponding exposure as the reference and were displayed within the 1st to 99th percentile range of the exposure distribution. Abbreviations: AIC, Akaike information criterion; RCS, restricted cubic spline.

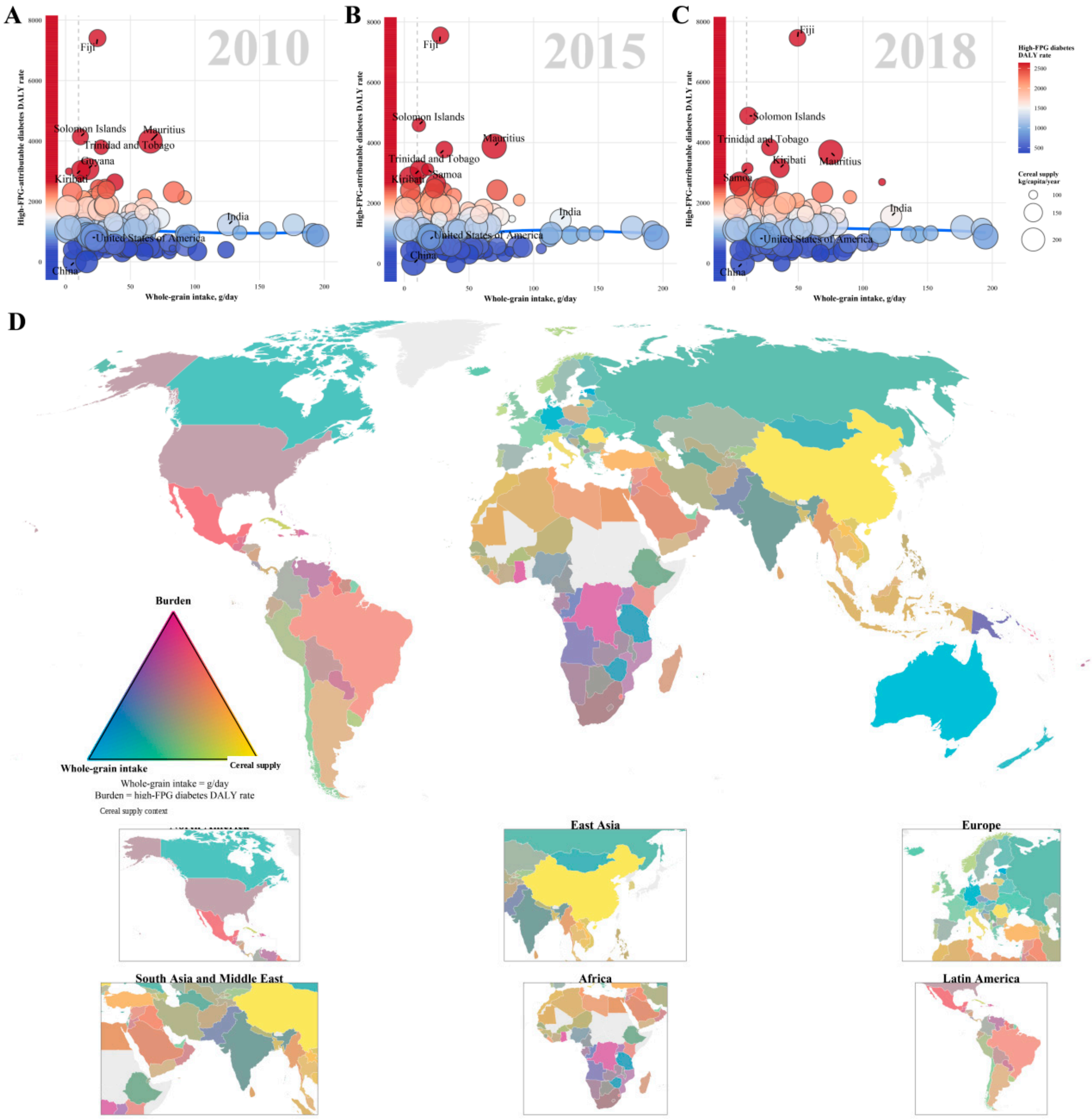

Figure S4. Global distribution of country-level whole-grain intake, high-FPG-attributable diabetes burden, and cereal supply context. This figure shows whole-grain intake, high-FPG-attributable diabetes DALY rates, and cereal supply context for 163 countries in 2010, 2015, and 2018. A–C, two-dimensional distributions of country-level whole-grain intake, high-FPG-attributable diabetes burden, and cereal supply; D, geographic distribution of these country-level dimensions in 2018. DALY, disability-adjusted life-year; FAOSTAT, Food and Agriculture Organization Corporate Statistical Database; FPG, fasting plasma glucose; GBD, Global Burden of Disease Study; GDD, Global Dietary Database.

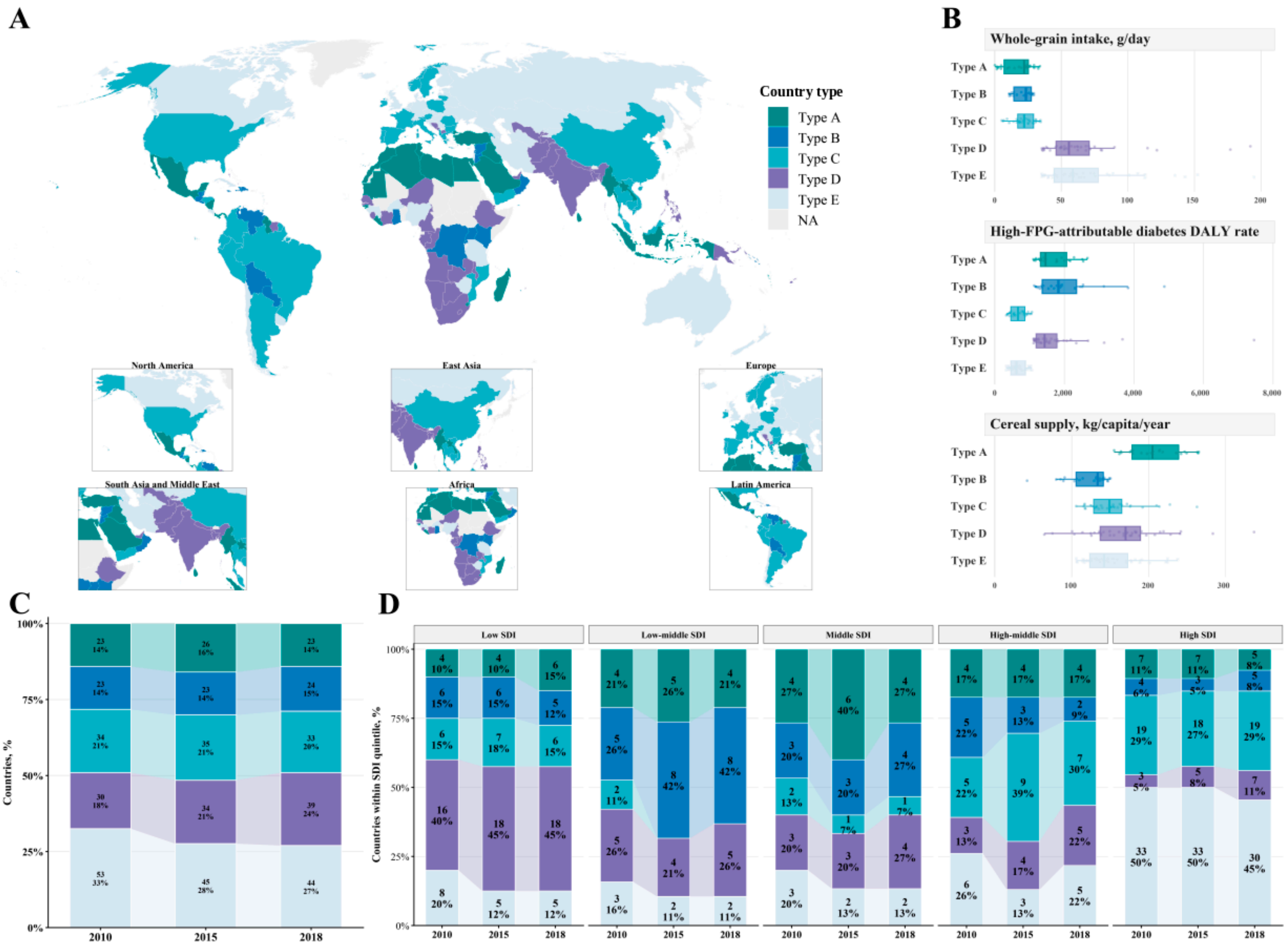

Figure S5. Country-level descriptive typology based on whole-grain intake, high-FPG-attributable diabetes burden, and cereal supply context. This figure shows the country-level typology constructed using medians from pooled country-year observations in 2010, 2015, and 2018. A, geographic distribution of country types in 2018; B, quantitative comparison of the three dimensions across country types; C, changes in typology composition across years; D, distribution by Socio-demographic Index category. SDI, Socio-demographic Index.
